# Predicting HIV viral non-suppression in Uganda: development and validation of machine learning and risk stratification models using routine EMR data

**DOI:** 10.3389/frai.2026.1869992

**Published:** 2026-07-16

**Authors:** Maria Magdalene Namaganda, Stathis Gennatas, Laura Merson, Esteban Garcia, Tom Edinburgh, Joyce Nakatumba Nabende, David Patrick Kateete, Charles Batte, Misaki Wayengera, Daudi Jjingo, Edgar Kigozi, Gerald Mboowa

**Affiliations:** 1 Department of Immunology and Molecular Biology, School of Biomedical Sciences, College of Health Sciences, Makerere University, Kampala, Uganda; 2 Institute for Global Health Sciences, University of California, San Francisco, San Francisco, CA, United States; 3 International Severe Acute Respiratory and Emerging Infectious Consortium, Pandemic Sciences Institute, University of Oxford, Oxford, United Kingdom; 4 CareerBiome Foundation, Kampala, Uganda; 5 Department of Computer Science, School of Computing and Information Technology, Makerere University, Kampala, Uganda; 6 Lung Institute, School of Medicine, College of Health Sciences, Makerere University, Kampala, Uganda; 7 African Center of Excellence in Bioinformatics and Data-Intensive Sciences, Makerere University, Kampala, Uganda; 8 Makerere University Biomedical Research Center, School of Biomedical Sciences, College of Health Sciences, Makerere University, Kampala, Uganda

**Keywords:** antiretroviral therapy, decision curve analysis, electronic medical records, explainable artificial intelligence, HIV viral non-suppression, machine learning, risk stratification, Uganda

## Abstract

**Background:**

Viral non-suppression is the primary actionable risk state in routine HIV care, yet most individuals are identified after virological failure and/or drug resistance, rather than proactively. In Uganda and similar resource-limited settings, routine electronic medical records (EMR) are collected at scale but remain underused for targeted, data-enabled risk stratification. We aimed to develop and internally validate machine learning and regularized regression models for predicting viral non-suppression using routine monitoring data.

**Methods:**

We developed and internally validated prediction models for viral non-suppression (viral load ≥1,000 copies/mL) using routinely recorded EMR variables from the TASO Uganda open cohort (2014–2024; *n* = 33,384). Twenty variables were used across four models: logistic regression (LR), elastic net regularized logistic regression (ENET), random forest (RF), and extreme gradient boosting (XGB), evaluated on a stratified 80:20 test set. Precision-recall AUC (PR-AUC) was the primary metric; ROC-AUC, Brier score, and decision curve analysis were assessed; bootstrap 95% CIs (2,000 replicates) were computed for discrimination metrics.

**Results:**

On the test set (*n* = 6,677), RF achieved PR-AUC 0.248 (95% CI 0.207–0.291) and ROC-AUC 0.758 (0.732–0.780); ENET achieved PR-AUC 0.237 (0.198–0.279) and ROC-AUC 0.750 (0.726–0.772); confidence intervals overlapped across all four models. RF and ENET achieved Brier scores of 0.055 (8% below the null of 0.060) and maximum net benefit of 0.055 in decision curve analysis. At the capacity-first threshold (top 5% predicted risk), RF flagged 325 individuals (4.9%; PPV 0.338, NPV 0.949) and ENET flagged 334 (5.0%; PPV 0.305, NPV 0.948). Current ART class (PI-based: OR 11.07; NNRTI-based: OR 4.80), poor adherence (OR 7.71), TB history (OR 2.08), and male sex (OR 1.62) were the strongest predictors.

**Conclusions:**

Routine EMR data support meaningful, calibrated viral non-suppression risk prediction across a large, multi-site Ugandan HIV program. At a capacity-first threshold, both models achieved approximately five-fold enrichment over background prevalence, with clinical utility confirmed by decision curve analysis. Prospective external validation and workflow integration are required before deployment.

## Introduction

Viral load monitoring is the cornerstone of contemporary HIV treatment programs, providing the definitive measure of treatment response and the earliest reliable signal of treatment failure. While viral load testing is the standard for monitoring antiretroviral therapy, it is one component of a broader clinical management toolkit that also includes CD4 count monitoring, clinical staging, adherence assessment, and opportunistic infection screening. In clinical practice, however, viral non-suppression is not primarily a diagnostic endpoint, it is an actionable risk state that signals modifiable barriers to sustained suppression: inconsistent adherence, suboptimal regimens, pharmacokinetic interactions, interruptions in care, or structural vulnerabilities that hinder continuous engagement with treatment (UNAIDS, 2023a; World Health Organisation, 2021). The clinical and programmatic value of viral load monitoring therefore depends not only on the capacity to detect non-suppression after it occurs, but on the ability to identify individuals most likely to develop it in sufficient time to intervene preventively.

In Uganda and across Sub-Saharan Africa, this proactive capacity is constrained by two converging realities. Clinical teams operate under significant workload pressure, with patient-to-clinician ratios that rarely permit individualized, longitudinal risk assessment for every person on antiretroviral therapy (ART) (Kiragga et al., 2019; UNAIDS, 2023a; Zakumumpa et al., 2016). In addition, even where viral load monitoring infrastructure has expanded substantially under PEPFAR and Global Fund investment, the gap between a viral load being drawn and clinical action being taken, the viral load response cascade, remains prolonged in many settings (Boeke et al., 2021; Kippen et al., 2024; Shah et al., 2025). These donor investments face increasing sustainability pressures, emphasizing the importance of mobilizing local or national funds as well as maximizing the clinical yield of existing infrastructure through data-driven prioritization strategies (Fauci and Eisinger, 2018; TASO, n.d.; UNAIDS, 2023b). The practical consequence is that non-suppression is frequently identified and addressed reactively, after a period of undetected viraemia, rather than pre-empted through targeted engagement with those most at risk.

Risk prediction models trained on routinely collected program data offer a practical complement to routine viral load monitoring, not a replacement for it. In Uganda and similar settings, WHO guidelines recommend viral load testing every 6 to 12 months for stable adults on ART (WHO, 2023); this interval means non-suppression risk may evolve between scheduled tests without a systematic mechanism to identify those most likely to be non-suppressed at their next test. If an individual's clinical and demographic record contains sufficient signal to flag elevated non-suppression risk before a new viral load result is available, that signal can be used to trigger targeted interventions within existing service capacity, including intensified adherence counseling, earlier clinical review, or expedited viral load testing, as a complement to routine viral load monitoring (Bisaso et al., 2018; Mamo et al., 2023).

Routine EMR systems now capture individual-level data at scale across HIV programs in Uganda and the broader East African region, encompassing demographic information, ART history, clinical staging, laboratory results, adherence records, and, in program settings such as The AIDS Support Organization (TASO) Uganda, selected psychosocial and socioeconomic fields (Namaganda et al., 2026; Ndisha et al., 2023; Zhang et al., 2023). Routine EMR data are available at population scale, updated continuously and require minimal research infrastructure to access (Boeke et al., 2021).

The majority of published HIV prediction research from Sub-Saharan Africa has relied on research-grade datasets, including cohort studies with high predictor completeness, genotypic resistance testing, or prospectively collected adherence measures (Kagendi and Mwau, 2023). While scientifically informative, models trained on such datasets may overestimate real-world predictive signal, because the predictor types and completeness levels available in research settings are rarely replicated in routine service delivery. A model trained on routinely collected, operationally representative data, with missingness handled explicitly and viral-load-derived predictors excluded, offers a more deployable and reliable estimate of what prediction from routine EMR can actually achieve (Steyerberg, 2019).

There is also a broader rationale for embedding prediction within the EMR rather than in a separate research tool. Integration with existing clinical systems means that risk scores can be generated without additional data collection, and that clinician-interpretable predictor associations and patient-level risk drivers can be embedded within the existing review workflow (Kimaina et al., 2020). The TASO program context, data structure, and programmatic rationale for this approach are described in the companion perspective paper (Namaganda , 2026).

A growing literature has examined machine-learning and statistical prediction of virological outcomes in Sub-Saharan African HIV programs, with reported ROC-AUC values spanning 0.65 to 0.82 across studies (Ahmed et al., 2019; Gelaw et al., 2021; Kagendi and Mwau, 2023). In Uganda specifically, Ngema and colleagues demonstrated the feasibility of XGBoost-based non-suppression prediction in a single-facility cohort (*n* = 1,101), achieving ROC-AUC of 0.80 with adherence as the dominant predictor (Ngema et al., 2026). Our companion systematic review and meta-analysis of predictors of virological failure in East Africa (2016–2023; 29,829 participants) estimated a pooled non-suppression prevalence of 19.4% and identified consistent evidence that poor adherence, advanced HIV disease, TB co-infection, male sex, and older ART regimen classes are predictive across settings; findings that directly informed our predictor selection (Namaganda et al., 2025).

This study addresses whether predictive signal from routine program data is robust to the multi-site heterogeneity, variable data quality, and evolving treatment landscapes of a large national program setting. Single-site, research-grade cohorts provide important proof-of-concept evidence but differ systematically from the contexts in which a deployed tool would need to perform (Riley et al., 2019). Beyond scale, this study addresses four methodological gaps not consistently addressed in the existing literature. First, confidence intervals around discrimination metrics are rarely reported, making it impossible to judge whether observed differences between model classes are statistically meaningful. Second, calibration—the degree to which predicted probabilities align with observed outcome rates, is a prerequisite for threshold-based clinical decision-making but is seldom assessed alongside discrimination (Van Calster et al., 2019). Third, clinical utility via decision curve analysis is largely absent from the HIV prediction literature, leaving the question of whether model-guided prioritization improves on uniform strategies unaddressed. Fourth, pre-specified operational operating points that translate continuous predicted probabilities into concrete program workflow decisions have rarely been provided.

The AIDS Support Organization (TASO) Uganda was established in 1987 and is the country's oldest and largest indigenous HIV service organization. It operates 11 Centers of Excellence spanning geographically and programmatically distinct urban and rural catchments across all four major regions of the country: northern Uganda (Gulu), eastern Uganda (Jinja, Mbale, Tororo, Soroti, Masindi), western Uganda (Mbarara, Rukungiri), and central/southern Uganda (Entebbe, Mulago, Masaka). These sites serve populations with substantially different HIV epidemiological profiles, socioeconomic conditions, and levels of health system access, making the program an unusually representative sample of Uganda's HIV treatment landscape. TASO operates as an independent non-governmental organization delivering comprehensive HIV care, treatment, and prevention services in a complementary relationship with Uganda's Ministry of Health, operating within the same national ART guidelines and supply chain, but maintaining its own structured clinical data infrastructure and psychosocial service model. TASO receives funding from PEPFAR, the Global Fund, and other international partners, and serves a substantial proportion of people living with HIV in Uganda; it however does not encompass all HIV treatment sites in Uganda, as some of PLHIV in Uganda receive care through Ministry of Health public facilities.

The TASO EMR registry spans decades of routine service delivery that encompasses policy transitions including; the scale-up of viral load testing, Test-and-Treat policy, and the progressive national rollout of integrase strand-transfer inhibitor (INSTI) based first-line therapy from 2018 onwards (Ministry of Health, 2020; TASO, n.d.). This policy transition from non-nucleoside reverse transcriptase inhibitor (NNRTI) to INSTI-dominated first-line therapy (Phillips et al., 2020; Vitoria et al., 2018), created a distributional shift in the client population and treatment landscape that directly bears on model performance and temporal generalizability, as described in the companion descriptive cohort paper (Namaganda et al., 2026; WHO, 2023). TASO registry captures not only standard demographic and ART history variables but also routine indicators of clinical vulnerability and service delivery engagement; adherence categories, nutritional status, comorbidities, and selected psychosocial and socioeconomic fields that are directly relevant to non-suppression risk but frequently absent from public-sector EMR systems (Namaganda et al., 2026; TASO, n.d.).

## Study objectives

This study had four specific objectives. First, to develop and internally validate prediction models for viral non-suppression using a, deployment- feasible set of routinely available TASO EMR predictors, with exclusion of viral-load-derived variables to prevent data leakage. Second, to evaluate model performance across three complementary domains; discrimination, calibration, and clinical utility via decision curve analysis; with bootstrap confidence intervals to support honest comparative statements. Third, to provide a two-layer explainability framework, comprising clinician-interpretable ENET odds ratios and RF SHAP global and local importance analyses, to identify the principal drivers of non-suppression risk. Fourth, to characterize two pre-specified, operationally grounded operating points; a screening-first and a capacity-first threshold that translate continuous predicted probabilities into actionable program-level decisions, and to assess model transportability across TASO sites and robustness to temporal distribution shift.

The intended use is pragmatic risk stratification within routine HIV care: to support targeted adherence reinforcement, earlier clinical review and prioritized viral load follow-up for individuals most likely to be non-suppressed, within the real capacity constraints of the TASO program and similar resource-limited settings.

## Materials and methods

### Ethics statement

This study was conducted as a secondary analysis of de-identified routine clinical records from TASO Uganda. Ethical approval was granted by the School of Biomedical Sciences Research Ethics Committee (SBS-REC), Makerere University (reference: SBS-2024-539), which waived the requirement for informed consent on the grounds that the analysis involved retrospective review of de-identified electronic records with no direct participant contact. Additional approvals were obtained from the Uganda National Council for Science and Technology (UNCST; reference: HS3982ES) and TASO administrative clearance (TASO REC/ADMC11/2024-UG-REC-009). All direct identifiers were replaced with study identifiers prior to analysis; the re-identification key remained securely held by TASO and was not accessible to the analysis team. Data abstraction from the source registry occurred on 10 December 2024.

This study is reported in accordance with the TRIPOD-AI reporting guideline for prediction model development and validation studies (Collins et al., 2024). A completed TRIPOD-AI checklist is provided as Supplementary File S1.

### Study design and setting

We developed and internally validated prognostic prediction models using retrospectively collected program data from the TASO Uganda open cohort (2014–2024; 11 sites). The analytic dataset was person-level: each record represented one individual, with predictor columns capturing demographic, clinical, treatment, and follow-up information accumulated during cohort observation. The prediction target was each individual's most recent recorded viral load outcome, providing one binary endpoint per person and aligning the modeling task with the patient-level dataset structure. Predictors were restricted to non-viral-load EMR variables available at the point of routine clinical review, reflecting the intended prospective clinical application: estimating non-suppression risk from a patient's current profile before a new viral load result is obtained. TASO operates 11 Centers of Excellence spanning urban and rural catchments (Entebbe, Gulu, Jinja, Masaka, Masindi, Mbale, Mbarara, Mulago, Rukungiri, Soroti, Tororo). Full cohort structure and program context are described in the companion cohort paper (Namaganda et al., 2026) and summarized here only insofar as they directly bear on model development and the interpretation of predictor availability.

### Cohort and analytic sample

The study cohort spans 1 January 2014 to 31 October 2024 and comprised 54,348 people living with HIV across 11 TASO sites after exclusion of records with implausible ART initiation dates. The prediction dataset was restricted to individuals with an observed most-recent viral load, defining the analytic cohort (*n* = 33,384; 61.4% of the parent registry; 61.8% female and 38.2% male). The cohort was partitioned using a stratified 80:20 random split; training set *n* = 26,707, held-out test set *n* = 6,677; stratified on the outcome to preserve non-suppression prevalence. The split was performed once with fixed seed 2,601 and not modified at any stage. All preprocessing, tuning, and model fitting were performed within the training set; the test set was accessed once for final performance evaluation.

### Outcome definition

The prediction target was viral non-suppression (NS) at the most recent recorded viral load, defined as ≥1,000 copies/mL consistent with national and WHO treatment monitoring guidelines (Ministry of Health, 2020; WHO, 2022). Viral suppression (VS) was defined as < 1,000 copies/mL. We modeled non-suppression rather than confirmed virological failure because repeat confirmatory testing and failure-confirmation fields were not uniformly available in routine records. Throughout this manuscript, “failure” and “success” appear only as technical identifiers in the source data and analysis code.

### Predictors and feature set

Predictors were restricted to a pre-specified (Collins et al., 2015), deployment-feasible Feature Set A, locked prior to modeling under three criteria: (1) deployment feasibility: routinely recorded in the TASO EMR without additional data collection; (2) leakage avoidance (Kaufman et al., 2012): all viral-load-derived predictors excluded, as these would be unavailable at the point of clinical review before a new result is obtained; (3) clinical breadth: variables spanning five domains identified in the regional literature and our companion systematic review and meta-analysis (Namaganda et al., 2025).

Feature Set A comprised 20 variables—demographic/administrative: health site, sex, current age, age at ART initiation, ART start year; treatment history: duration on ART, baseline and current ART class; immuno-clinical: baseline CD4 count, WHO stage, BMI; comorbidities: nutritional status, TB history, diabetes, hypertension; psychosocial/socioeconomic: adherence category, marital status, education, employment, psychosocial support status. ART start date and viral load dates were retained solely as partitioning variables for the temporal sensitivity analysis.

### Preprocessing and handling of missing data

Variable-level availability was characterized in the training data and classified as routinely available ( ≤ 5% missing), moderately available (5–20%), or sparsely available (>20%), reported in Supplementary Table S1. Predictor distributions in the training set are reported in Supplementary Table S2. All preprocessing steps were estimated exclusively on training data and applied unchanged to the held-out test set, ensuring that no information from the test set influenced preprocessing (Hastie et al., 2009). The pipeline, implemented as a single shared recipe applied uniformly across all candidate model classes, comprised the following sequential steps. Binary missingness indicators were created for each numeric predictor to encode the fact that a value was absent, independently of subsequent imputation. Missing values in nominal predictors were assigned a dedicated “unknown” category (Little, 2019). Novel categorical levels absent from the training data were assigned a separate “novel” category to prevent prediction-time errors. Infrequent categorical levels representing fewer than 1% of training observations were collapsed into an “other” category to reduce sparsity (Kuhn and Johnson, 2019). Numeric predictors were imputed using the training-set median; nominal predictors using the training-set mode. Nominal predictors were one-hot encoded to produce a fully numeric design matrix applicable to all candidate model classes. Zero-variance predictors created by the encoding steps were removed.

### Candidate model classes

Four model classes were evaluated, selected to span a range of complexity and interpretability, from a transparent linear baseline to non-linear ensemble approaches.

Logistic regression (LR) was included as a simple, transparent clinical comparator with no regularization and no hyperparameter tuning.

Elastic net regularized logistic regression (ENET) was included as a candidate model *a priori* and was subsequently selected as the clinical baseline after joint evaluation of all four models on calibration and clinical utility. ENET retains the familiar logistic regression framework while handling multicollinearity and shrinking unstable coefficients through a combination of L1 and L2 penalties. Two hyperparameters were tuned: overall regularization strength (penalty) and the L1-to-L2 mixing ratio (mixture), over a space-filling Latin hypercube grid of 25 candidate combinations (Zou and Hastie, 2005).

Random forest (RF) was evaluated as a non-linear ensemble model capable of capturing predictor interactions without explicit specification. The number of candidate predictors at each tree split (mtry, range 2–30) and minimum node size (min_n, range 5–80) were tuned over a space-filling Latin hypercube grid of 20 combinations, with 300 trees. Permutation-based variable importance was computed for explainability purposes (Breiman, 2001).

Extreme gradient boosting (XGB) was evaluated as a second non-linear ensemble model. Seven hyperparameters were jointly tuned over a space-filling grid of 25 combinations: number of trees (300–1,200), tree depth, learning rate, minimum loss reduction for a split, subsample proportion, number of candidate predictors per split, and minimum node size. To address class imbalance, XGB was fitted with a scale-positive-weight parameter (McKay et al., 1979) equal to the ratio of virally suppressed to non-suppressed observations in the training data (approximately 14.6) (Chen and Guestrin, 2016).

All models used the same preprocessing recipe and were embedded in unified workflows, ensuring that preprocessing was fitted on training data and applied identically at prediction time.

### Hyperparameter tuning and model selection

Hyperparameter tuning and internal validation used 5-fold stratified cross-validation (Kohavi, 1995) within the training set, with precision-recall AUC (PR-AUC) as the primary tuning metric. PR-AUC was chosen over ROC-AUC because the outcome was rare (non-suppression prevalence of 6.4%). For imbalanced outcomes, PR-AUC is more sensitive to model performance on the minority class, as ROC-AUC can remain high even when positive-class prediction is poor (Saito and Rehmsmeier, 2015). The configuration achieving the highest cross-validated PR-AUC was selected for each tunable model class; final workflows were refit on the complete training set and applied once to the held-out test set. Final canonical model selection was based on joint evaluation of discrimination, calibration, and clinical utility as described in the Results section.

## Performance evaluation on the held-out test set

### Discrimination

Discrimination was quantified using PR-AUC as the primary metric (Davis and Goadrich, 2006) and ROC-AUC as a secondary metric to allow comparison with the existing literature. For PR-AUC, the null expectation for a non-informative classifier equals the outcome prevalence (0.064 for this study); for ROC-AUC, the null expectation is 0.50. Bootstrap 95% confidence intervals were computed using 2,000 replicates with patient-indexed sampling and seed 2601.

### Calibration

Calibration was assessed using the Brier score (Brier, 1950), defined as the mean squared error of predicted probabilities against observed outcomes, reported alongside a null reference (*p* × [1–p], where *p* = NS prevalence). For calibration plots, test-set observations were grouped into ten equal-sized bins by predicted risk, and mean predicted probability was plotted against observed NS rate within each bin; systematic deviation from the diagonal indicates miscalibration.

### Clinical utility

Clinical utility was assessed using decision curve analysis (DCA), in which net benefit was calculated across threshold probabilities from 0.01 to 0.20 (Vickers and Elkin, 2006), compared against treat-all and treat-none reference strategies. Maximum net benefit was the primary DCA summary statistic.

### Threshold-based operating points

Two threshold-based operating point types were pre-specified prior to modeling to translate continuous predicted probabilities into operational clinical decisions (Pauker and Kassirer, 1980).

Screening-first threshold: defined as the minimum predicted risk threshold at which the model achieved a sensitivity of at least 0.95 in the training data. This threshold reflects a use case in which missing a true non-suppressed individual carries a high cost and the program is willing to accept broader flagging in exchange for near-complete case capture.

Capacity-first threshold: the predicted risk value at the 95th percentile of the training-set risk distribution, corresponding to the top 5% of individuals by predicted risk. This reflects a capacity-constrained clinical environment in which only a small proportion of individuals can be prioritized for intensified review within a given period.

For each operating point and model, performance on the held-out test set was reported as sensitivity, specificity, positive predictive value (PPV), negative predictive value (NPV), the number of individuals flagged, and the proportion of the test set flagged.

### Explainability

Explainability was implemented for the two canonical models.

ENET: refit logistic regression for odds ratios. Feature Set A variables were one-hot encoded, producing an expanded numeric design matrix. Features with non-zero ENET coefficients at the selected penalty value were identified and re-entered into an unpenalized logistic regression on the baked training matrix to yield interpretable odds ratios; regularized ENET coefficients are shrunk toward zero and cannot be directly exponentiated. Events-per-variable adequacy was assessed against the recommended minimum of 10 (Riley et al., 2019).

RF: SHAP-based global and local explanations. SHAP (SHapley Additive exPlanations) values were estimated via Monte Carlo integration using the fastshap package, as exact TreeSHAP is unavailable for ranger-fitted forests. Global feature importance was computed on 1,500 held-out test-set observations over 50 replicates (seed 2601); mean absolute SHAP values ranked predictors by average contribution. Local SHAP profiles were generated for the three highest-risk test-set individuals (Lundberg and Lee, 2017).

Partial dependence plots (PDPs) and individual conditional expectation (ICE) plots were produced for selected numeric predictors to characterize predictor-risk relationships.

## Sensitivity analyses

### Site-held-out transportability

To assess whether model performance generalized across TASO service settings, a leave-one-site-out cross-validation (Debray et al., 2013) was performed using the training data. For each of the 11 TASO sites in turn, models were trained on data from all remaining sites and evaluated on the held-out site, using the same preprocessing pipeline and best-performing hyperparameters from the primary analysis. This analysis assesses transportability within the TASO network; it does not constitute full external validation, which would require independent data from sites outside the TASO system.

### Temporal split sensitivity analysis

A temporal split sensitivity analysis assessed model robustness to the period- specific distribution shifts (Altman and Royston, 2000). The analytic dataset was partitioned by viral load date rather than at random, with the earlier records assigned to training and the later records to testing; the date threshold was set at the 80th percentile of the viral load date distribution. Viral load date was used only for partitioning and was not included as a predictor. Results are reported in Supplementary Table S5 and treated as supplementary given uncertainty in viral load date capture across sites.

### Software and reproducibility

All analyses were performed in R (version 4.4). Model development used the tidymodels framework (recipes, parsnip, tune, yardstick). Random forest models were fitted using the ranger engine; elastic net and logistic regression using glmnet; gradient boosting using xgboost. SHAP values were computed using the fastshap package. Decision curve analysis was implemented using a custom function equivalent to the standard net benefit formula, cross-referenced against the rmda package. A fixed random seed (2601) was applied at the start of the session. The 80:20 stratified split and all preprocessing parameters were fixed prior to modeling. The full analysis script is available as Supplementary material.

## Results

### Analytic cohort, outcome prevalence, and data split

**Figure 1:**
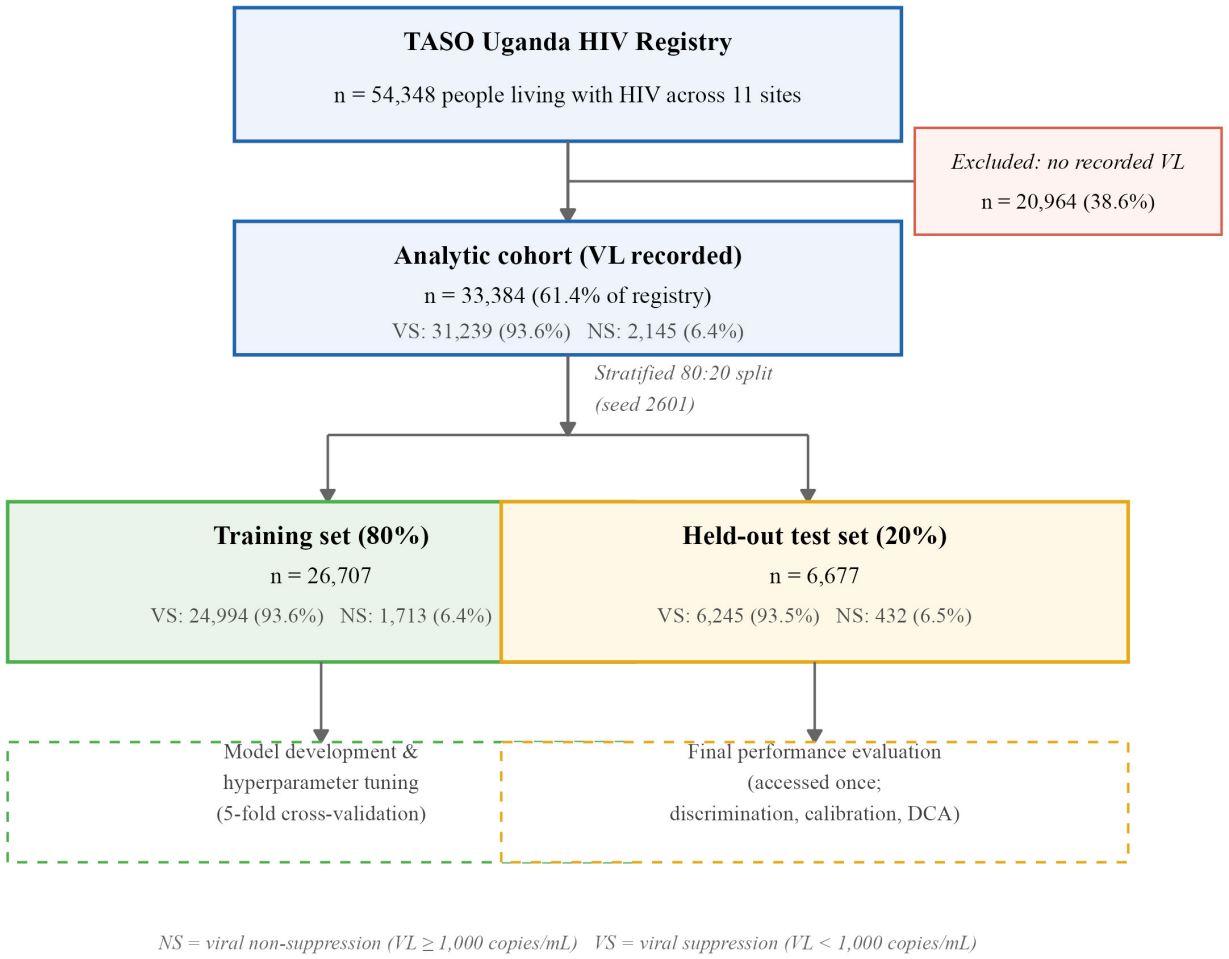
Derivation of the analytic cohort from the TASO Uganda HIV registry (2014–2024). Of 54,348 people living with HIV in the parent cohort, 33,384 (61.4%) had a recorded most-recent viral load result and were included in the prediction analysis. The 80:20 stratified random split (seed 2,601) preserved viral non-suppression prevalence at 6.4% in the training set and 6.5% in the held-out test set. NS, viral non-suppression (VL ≥1,000 copies/mL); VS, viral suppression (VL < 1,000 copies/mL); VL, viral load.

Of the 54,348 people living with HIV in the TASO cohort described in the companion descriptive paper; 33,384 (61.4%) had a recorded most-recent viral load result and were included in the prediction analysis. The remaining 20,964 individuals (38.6%) had no recorded viral load within the observation window and were excluded. The analytic cohort was 61.8% female (*n* = 20,615) and 38.2% male (*n* = 12,769). Within the analytic cohort, 2,145 individuals (6.4%) had viral non-suppression (VL ≥1,000 copies/mL) and 31,239 (93.6%) had viral suppression (VL < 1,000 copies/mL) (Figure 1, Table 1).

**Table 1:** Cohort derivation and analytic data partition, TASO Uganda HIV cohort (2014–2024).

Stage	*n*	%
Parent TASO cohort (Namaganda et al., 2026)	54,348	—
Included in prediction analysis (VL recorded)	33,384	61.4% of parent cohort
Excluded (no VL recorded)	20,964	38.6% of parent cohort
VS (VL < 1,000 copies/mL)	31,239	93.6% of analytic cohort
NS (VL ≥1,000 copies/mL)	2,145	6.4% of analytic cohort
Training set	26,707	80.0% of analytic cohort
— VS in training set	24,994	93.6% of training set
— NS in training set	1,713	6.4% of training set
Held-out test set	6,677	20.0% of analytic cohort
— VS in held-out test set	6,245	93.5% of held-out test set
— NS in held-out test set	432	6.5% of held-out test set

VS, viral suppression (VL < 1,000 copies/mL); NS, viral non-suppression (VL ≥ 1,000 copies/mL); VL, viral load. The 80:20 stratified random split (seed 2,601) preserved NS prevalence at 6.4% in the training set and 6.5% in the held-out test set.

The analytic cohort was divided into a training set of 26,707 individuals (80%) and a held-out test set of 6,677 (20%). Viral non-suppression prevalence was 6.4% in the training set (*n* = 1,713) and 6.5% in the held-out test set (*n* = 432), confirming that outcome frequency was preserved across the partition.

### Predictor availability in the analytic sample

Twenty predictors formed the pre-specified Feature Set A. Of these, 10 were recorded in at least 95% of training-set observations: health site, sex, current age, ART start year, and duration on ART were fully observed; current ART class and initiation age had fewer than 0.1% missing values; marital status (3.7%), baseline ART class (2.5%), and nutritional status (1.9%) were also near-complete. Eight predictors had moderate missingness (5–20%), including hypertension (6.2%), psychosocial support status (6.8%), diabetes (7.1%), baseline WHO stage (9.1%), employment status (10.6%), adherence category (10.8%), initial education (13.5%), and TB history (16.0%). Two predictors were sparsely recorded: baseline CD4 count was available in 35.1% of training records, and baseline BMI in 24.7% (Supplementary Table S1).

These patterns reflect variable data capture across clinical domains: core administrative and ART-related variables were reliably recorded at scale; clinical markers and psychosocial fields were less consistently documented across sites and time. After one-hot encoding of multi-level categorical predictors, the 20 Feature Set A variables expanded to approximately 80 binary and numeric terms in the baked design matrix; ENET regularization reduced this to 44 retained terms, which were re-entered into the refit logistic regression for interpretability. With 1,713 events in the training set and 44 retained predictors, the events-per-variable ratio was 39, well above the recommended minimum of 10 (Riley et al., 2019).

## Comparative performance of candidate models

Four candidate model classes were evaluated on the held-out test set (Table 2).

**Table 2:** Performance of all four candidate model classes on the held-out test set (n = 6,677; NS prevalence 6.5%).

Model	PR-AUC (95% CI)^a^	ROC-AUC (95% CI)^a^	Brier score	Max net benefit^b^
**RF**	^**^0.248 (0.207–0.291)^*^	^*^^**^0.758 (0.732–0.780)	^**^ **0.055**	**0.055**
XGB	0.241^c^	0.760^c^	0.063	0.027
**ENET**	^**^0.237 (0.198–0.279)^*^	^*^^**^0.750 (0.726–0.772)	^**^ **0.055**	**0.055**
LR	0.234 (0.195–0.276)	0.747 (0.723–0.769)	0.055	0.055
Null (marginal prevalence)^d^	0.064	0.500	0.060	—

^a^95% bootstrap confidence intervals (2,000 replicates, patient-indexed sampling, seed 2,601). Bold = retained canonical models (RF and ENET). ^b^Maximum net benefit from decision curve analysis across threshold range 0.01–0.20. ^c^95% bootstrap CIs not computed for XGB: XGB was excluded on calibration grounds (Brier 0.063 > null 0.060) prior to the bootstrap analysis. ^d^Null = a model that assigns the marginal NS prevalence to every observation. PR-AUC null equals the outcome prevalence. PR-AUC = precision–recall AUC; ROC-AUC. receiver operating characteristic AUC; ENET, elastic net; RF, random forest; XGB, extreme gradient boosting; LR, logistic regression. The symbol ^*^ denotes the retained models (RF and ENET) selected for primary analysis.

### Discrimination

**Figure 2:**
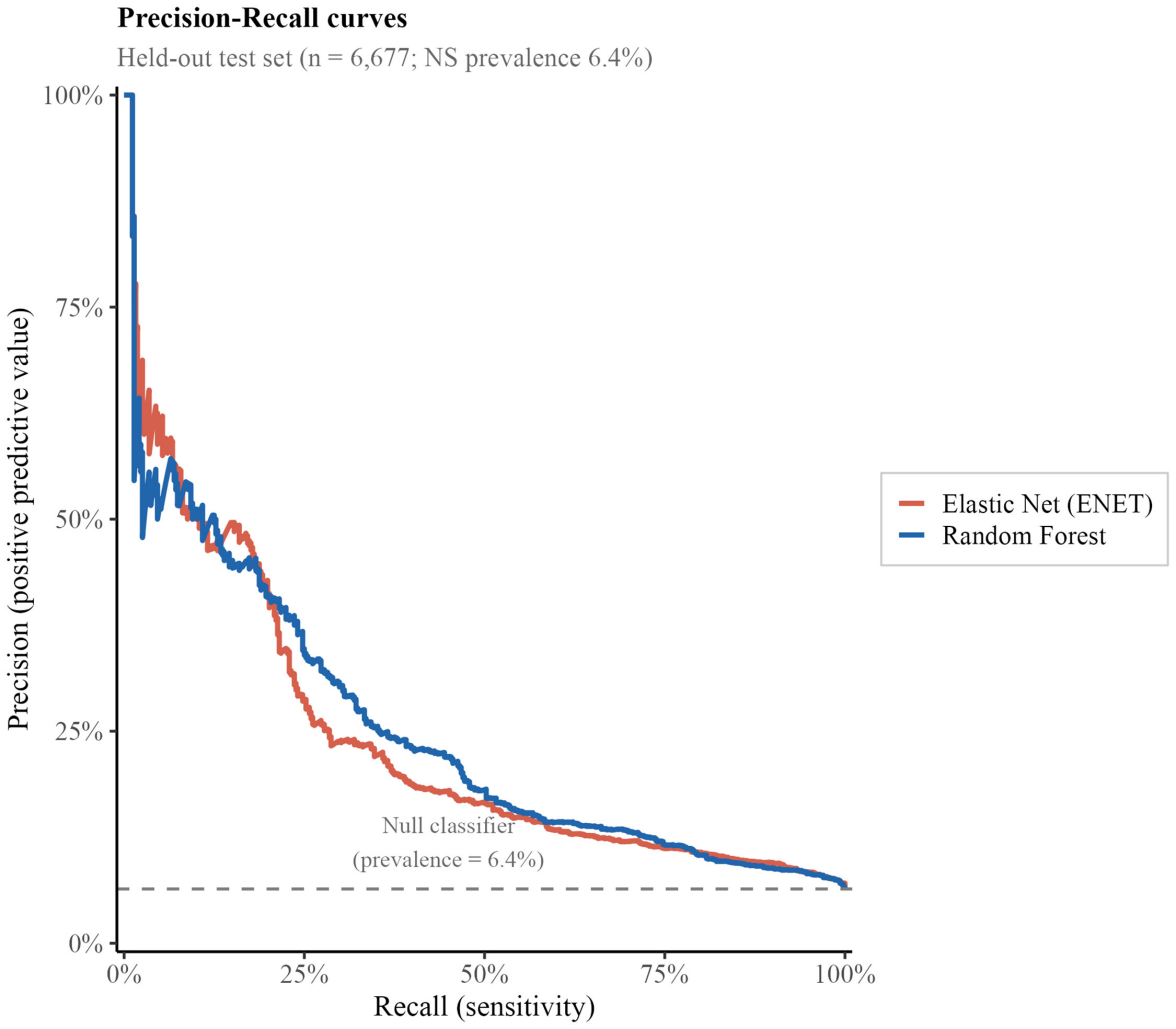
Precision-recall (PR) curve on the held-out test set (*n* = 6,677): precision (positive predictive value) plotted against recall (sensitivity) across all classification thresholds. The dashed horizontal line indicates the null classifier, whose precision equals the outcome prevalence (6.4%); a curve above this line indicates lift above chance. Area under the PR curve: RF 0.248 (95% CI 0.207–0.291); ENET 0.237 (95% CI 0.198–0.279).

**Figure 3:**
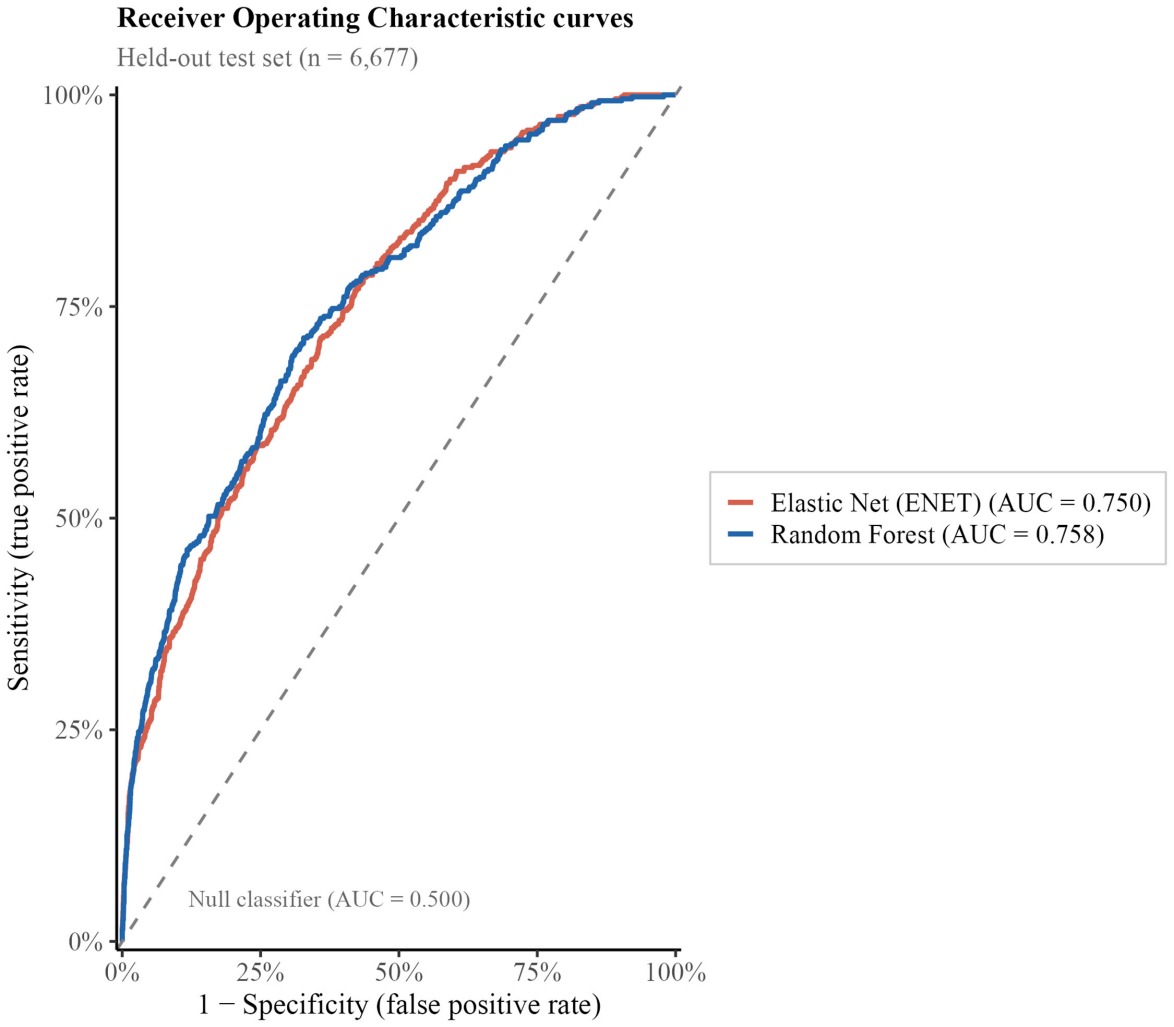
Receiver operating characteristic (ROC) curve on the held-out test set (*n* = 6,677): sensitivity plotted against 1–specificity. The dashed diagonal indicates the null classifier (ROC-AUC = 0.50). ROC-AUC: RF 0.758 (95% CI 0.732–0.780); ENET 0.750 (95% CI 0.726–0.772). Confidence intervals from 2,000 bootstrap replicates with patient-indexed sampling (seed 2,601). Both curves are plotted from held-out test set predictions only; training data were not used in evaluation. ENET, elastic net regularized logistic regression; RF, random forest.

**Figure 4:**
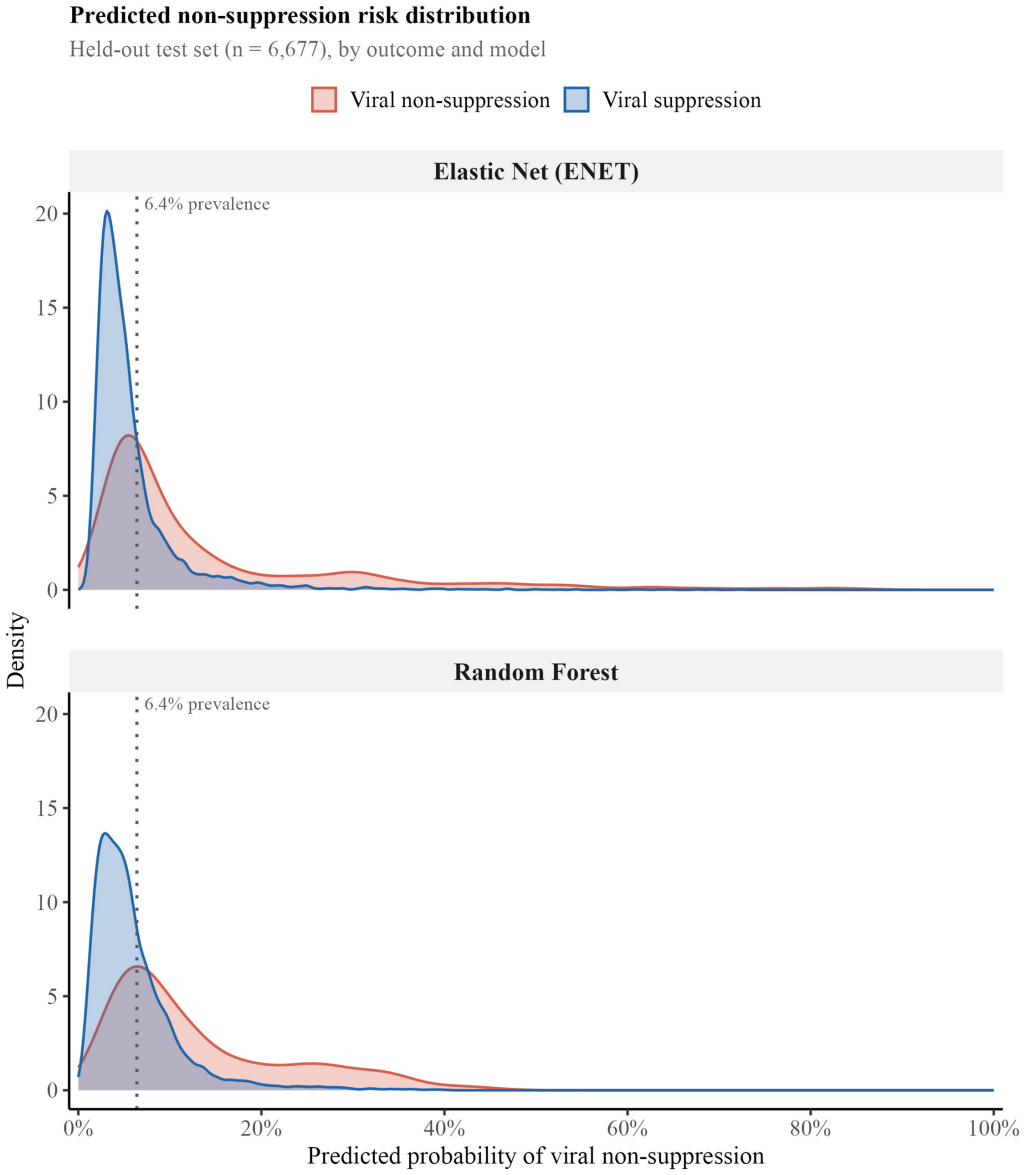
Distribution of predicted non-suppression risk by outcome group—held-out test set (*n* = 6,677). Density plots of model-assigned predicted probability of viral non-suppression, stratified by true viral load outcome, shown separately for Random Forest (upper panel) and Elastic Net (lower panel). Red: non-suppressed individuals (*n* = 432; 6.5%). Blue: virally suppressed individuals (*n* = 6,245; 93.5%). Greater rightward displacement of the non-suppressed distribution relative to the suppressed distribution indicates better model discrimination. The dotted vertical line marks the overall non-suppression prevalence (6.4%). Both panels are generated from held-out test set predictions only. ENET, elastic net regularized logistic regression; RF, random forest.

PR-AUC was highest for RF (0.248, 95% CI 0.207–0.291), followed by XGB (0.241), ENET (0.237, 95% CI 0.198–0.279), and LR (0.234, 95% CI 0.195–0.276). For reference, the expected PR-AUC of a non-informative classifier equals the outcome prevalence (0.064); all four models, therefore achieved meaningful lift above chance. On ROC-AUC, XGB ranked highest (0.760), followed by RF (0.758, 95% CI 0.732–0.780), ENET (0.750, 95% CI 0.726–0.772), and LR (0.747, 95% CI 0.723–0.769). Bootstrap 95% confidence intervals for PR-AUC and ROC-AUC overlapped across all models, indicating that the models cannot be statistically distinguished on discrimination alone. Bootstrap CIs were not computed for XGB, which was excluded on calibration grounds prior to the bootstrap analysis (Table 2, footnote c). PR and ROC curves for the retained models are shown in Figures 2, 3 respectively. The distribution of model-assigned predicted probabilities by outcome group is shown in Figure 4.

### Calibration

The null Brier score for a 6.4% prevalence outcome is 0.060. ENET, RF, and LR each achieved a Brier score of 0.055, representing an 8% improvement over the null. XGB performed worse than the null (Brier 0.063), indicating that its predicted probabilities were miscalibrated; a model that predicts the marginal event rate for every individual would outperform XGB on this metric.

### Clinical utility

**Figure 5:**
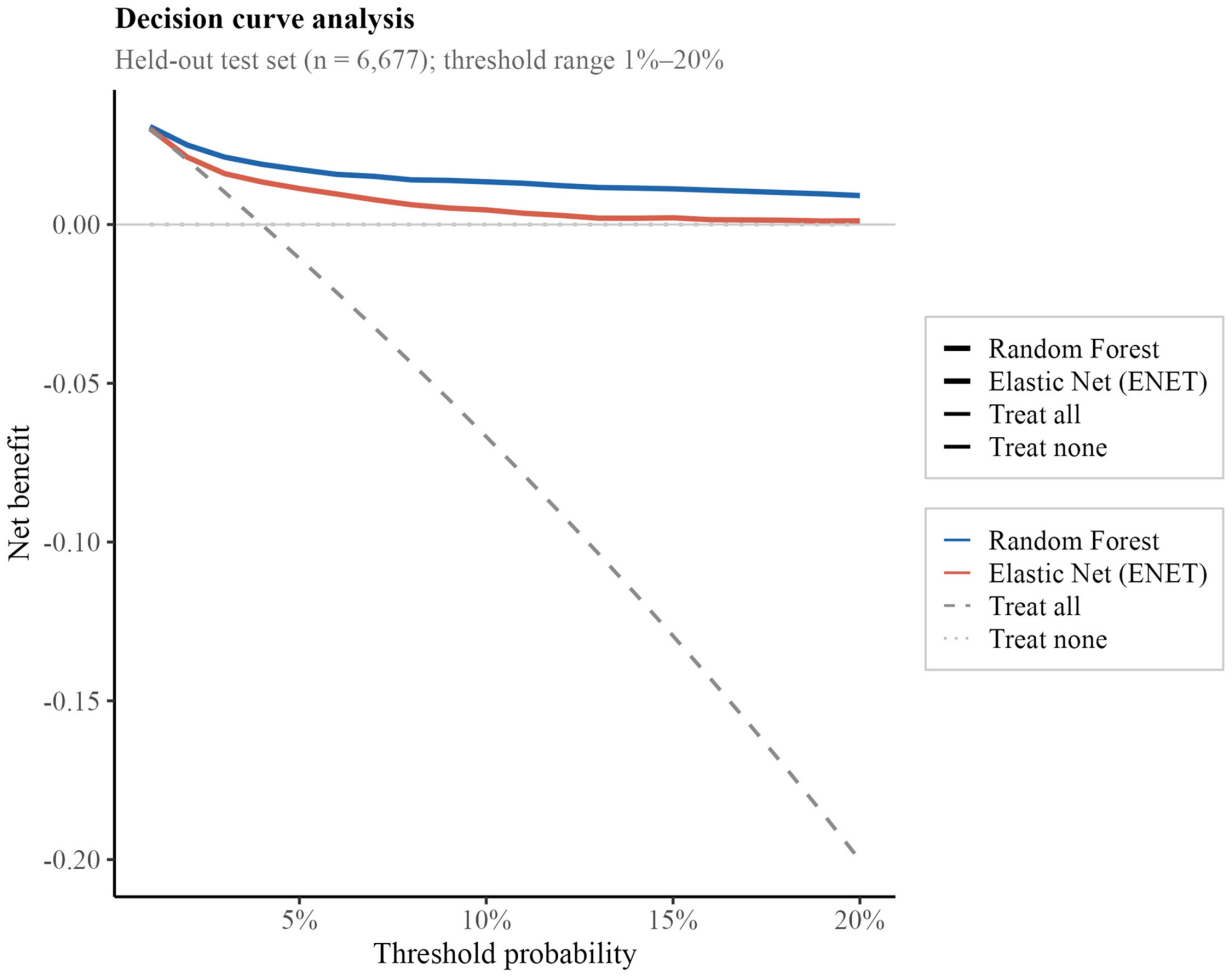
Decision curve analysis for ENET and RF on the held-out test set (*n* = 6,677), with treat-all and treat-none reference strategies. Net benefit is plotted across threshold probabilities 0.01–0.20. Both retained models provide net benefit above both reference strategies across this threshold range. XGB sustained no net benefit advantage over treat-all. Maximum net benefit: RF 0.055; ENET 0.055; XGB 0.027. ENET, elastic net regularized logistic regression; RF, random forest; XGB, extreme gradient boosting.

Decision curve analysis across threshold probabilities 0.01–0.20 showed that RF and ENET yielded net benefit above the treat-all and treat-none reference strategies, with maximum net benefit of 0.055 for both models. XGB sustained no net benefit advantage over treat-all across this threshold range (maximum net benefit 0.027; Figure 5, Table 2).

### Model selection

ENET was retained as the clinical baseline model and RF as the primary machine-learning model. Because bootstrap confidence intervals for PR-AUC overlapped across all models, model selection was not determined by discrimination alone; calibration and net benefit were decisive. XGB was not retained: its Brier score exceeded the null and it sustained no net benefit advantage over treat-all. RF was preferred over ENET as the primary model on the basis of its pre-specified SHAP explainability objectives and numerically higher PR-AUC point estimate. LR offered no gain over ENET on any metric and was not retained.

### Calibration and clinical utility of the retained models

**Figure 6:**
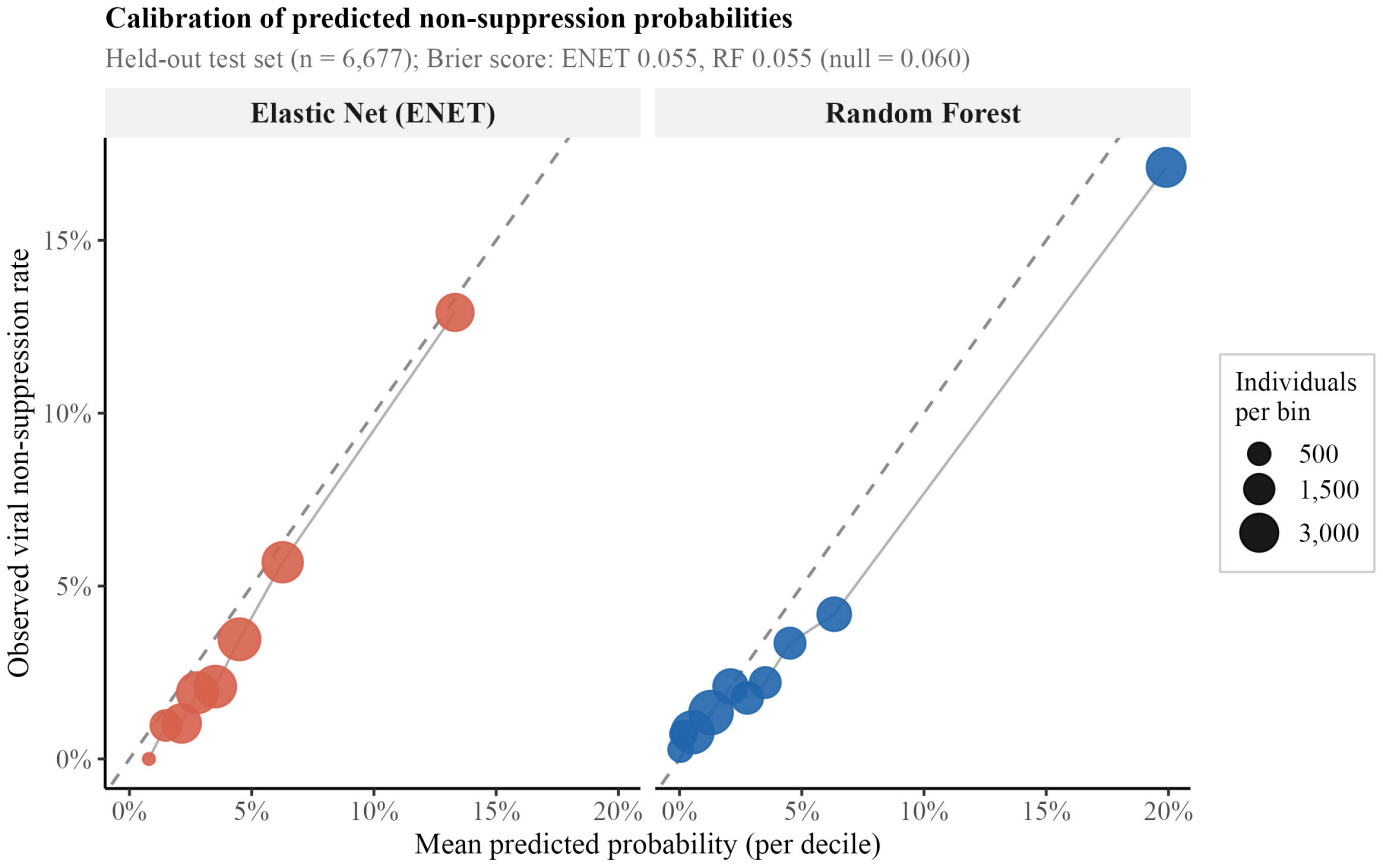
Calibration of ENET and RF predicted probabilities on the held-out test set (*n* = 6,677). Mean predicted risk plotted against observed viral non-suppression rate within deciles of predicted risk. The dashed diagonal indicates perfect calibration. Both ENET and RF show close agreement with the diagonal across the full predicted risk range, with no systematic over- or under-prediction. ENET, elastic net regularized logistic regression; RF, random forest.

On the held-out test set, ENET and RF showed closely matched and satisfactory calibration, both achieving Brier scores of 0.055, compared with a null of 0.060 (Figure 6). Calibration plots showed no systematic deviation from the diagonal across deciles of predicted risk for either model (Figure 6).

Decision curve analysis demonstrated that both ENET and RF provided net benefit above treat-all and treat-none strategies across threshold probabilities from 1 to 20%, with maximum net benefit reaching 0.055 for each model (Figure 5). The treat-all strategy yielded negative net benefit at thresholds above the 6.5% NS prevalence, reinforcing that model-guided prioritization produced better expected outcomes than blanket strategies in the clinically relevant range.

### Threshold-based operating characteristics

At the screening-first threshold (minimum threshold achieving ≥0.95 sensitivity in the training data), ENET flagged 6,676 of 6,677 individuals (99.99%; sensitivity 1.00, specificity < 0.001, PPV 0.065) and RF flagged all 6,677 (100%; sensitivity 1.00, specificity 0.00; Table 3). At a 6.5% NS prevalence, near-universal flagging is the necessary cost of achieving ≥95% sensitivity; this operating point is reported for completeness and is not a recommended implementation threshold.

**Table 3:** Threshold-based operating characteristics—ENET and RF, held-out test set (*n* = 6,677).

Model	Operating point	Threshold	Sensitivity	Specificity	PPV	NPV	Flagged *n*	Flagged %
ENET	Screening-first (sens ≥0.95)	0.008	1.000	< 0.001	0.065	1.000	6,676	99.99%
RF	Screening-first (sens ≥0.95)	0.004	1.000	0.000	0.065	—	6,677	100.00%
RF	Capacity-first (top 5%)	0.177	0.255	0.966	0.338	0.949	325	4.87%
ENET	Capacity-first (top 5%)	0.171	0.236	0.963	0.305	0.948	334	5.00%

Thresholds were selected from training-set predictions and applied without modification to the held-out test set. ENET, elastic net regularized logistic regression; PPV, positive predictive value; RF, random forest. PPV, positive predictive value; NPV, negative predictive value (not estimable for RF at the screening-first threshold, as all test-set individuals were flagged).

At the capacity-first threshold (top 5% of predicted risk in the training set), RF flagged 325 of 6,677 individuals (4.9%) with sensitivity 0.255, specificity 0.966, PPV 0.338, and NPV 0.949. ENET flagged 334 individuals (5.0%) with sensitivity 0.236, specificity 0.963, PPV 0.305, and NPV 0.948. The expected yield of non-suppressed individuals among those flagged is approximately 4.7- to 5.2-fold above the background prevalence of 6.5% (PPV 30.5–33.8%). Among those not flagged, the probability of maintained viral suppression exceeded 94.8% for both models.

## Predictors of viral non-suppression

### ENET refit logistic regression

To provide clinically interpretable predictor associations, the 44 ENET-selected features were re-entered into an unpenalised logistic regression on the baked training design matrix. Odds ratios and 95% confidence intervals from this refit model are reported in Table 4.

**Table 4:** ENET predictor associations with viral non-suppression—refit logistic regression. Predictors with OR >1.10 or <0.91, sorted by effect magnitude.

Predictor	OR	95% CI	*p*-value
Current ART class: PI-based	11.07	8.68–14.08	< 0.001
Adherence: poor/other	7.71	6.18–9.59	< 0.001
Current ART class: NNRTI-based	4.80	4.10–5.61	< 0.001
Current ART class: other	3.64	1.85–6.64	< 0.001
TB history: yes	2.08	1.67–2.57	< 0.001
Site: Mbale	1.65	1.39–1.94	< 0.001
Sex: male	1.62	1.45–1.80	< 0.001
Psychosocial support: undocumented	1.45	1.05–1.99	0.022
Employment: unemployed	1.25	1.09–1.43	0.001
Marital status: unmarried	1.16	1.04–1.30	0.009
Site: Rukungiri	0.69	0.53–0.87	0.003
Diabetes: yes	0.59	0.41–0.84	0.004
Site: Mbarara	0.58	0.46–0.71	< 0.001

Predictors with OR >1.10 or < 0.91, sorted by effect magnitude. Reference categories: ART class = INSTI-based; health site = Entebbe; sex = female; adherence = good; TB history = no; employment = employed; marital status = married/partnered; psychosocial support = documented. Odds ratios and 95% CIs are from an unpenalised logistic regression refit using the 44 ENET-selected predictors; regularized ENET coefficients cannot be directly exponentiated as odds ratios. Full 44-term table in Supplementary Table S4. ART, antiretroviral therapy; CI, confidence interval; ENET, elastic net regularized logistic regression; INSTI, integrase strand-transfer inhibitor; NNRTI, non-nucleoside reverse transcriptase inhibitor; OR, odds ratio; PI, protease inhibitor.

Current ART regimen class was the dominant predictor. Individuals on protease-inhibitor–based (PI-based) ART had markedly higher odds of non-suppression compared to those on integrase strand-transfer inhibitor (INSTI) based regimens (OR 11.07, 95% CI 8.68–14.08, *p* < 0.001). This association reflects a composite clinical signal that integrates current pharmacological regimen class with prior treatment history, prior virological failure, accumulated resistance pressure, and selection into a salvage therapy subgroup; the interpretive context is provided in the Discussion. Individuals on NNRTI-based ART also had substantially elevated odds (OR 4.80, 95% CI 4.10–5.61, *p* < 0.001), as did those on other non-INSTI classes (OR 3.64, 95% CI 1.85–6.64, *p* < 0.001). Collectively, remaining on any non-INSTI regimen was associated with substantially higher non-suppression risk relative to INSTI-based therapy.

Poor adherence was the second strongest predictor (OR 7.71, 95% CI 6.18–9.59, *p* < 0.001). In the TASO EMR, adherence category is recorded by a health worker as a categorical rating based on pill counts and the assessment of medication-taking behavior from clients report and refill timing (TASO). TB history was independently associated with non-suppression (OR 2.08, 95% CI 1.67–2.57, *p* < 0.001).

Male sex was associated with higher non-suppression odds than female sex (OR 1.62, 95% CI 1.45–1.80, *p* < 0.001).

Unmarried marital status (OR 1.16, 95% CI 1.04–1.30, *p* = 0.009), unemployment (OR 1.25, 95% CI 1.09–1.43, *p* = 0.001), and undocumented psychosocial support status (OR 1.45, 95% CI 1.05–1.99, *p* = 0.022) were each associated with higher non-suppression odds, pointing to the independent contribution of socioeconomic vulnerability.

Substantial between-site variation was present after adjustment for individual-level predictors. Compared to the reference site (Entebbe), individuals at Mbale had higher non-suppression odds (OR 1.65, 95% CI 1.39–1.94, *p* < 0.001), while Mbarara (OR 0.58, 95% CI 0.46–0.71, *p* < 0.001) and Rukungiri (OR 0.69, 95% CI 0.53–0.87, *p* = 0.003) had lower odds.

Diabetes was associated with lower non-suppression odds in the adjusted model (OR 0.59, 95% CI 0.41–0.84, *p* = 0.004).

### RF global feature importance

**Figure 7:**
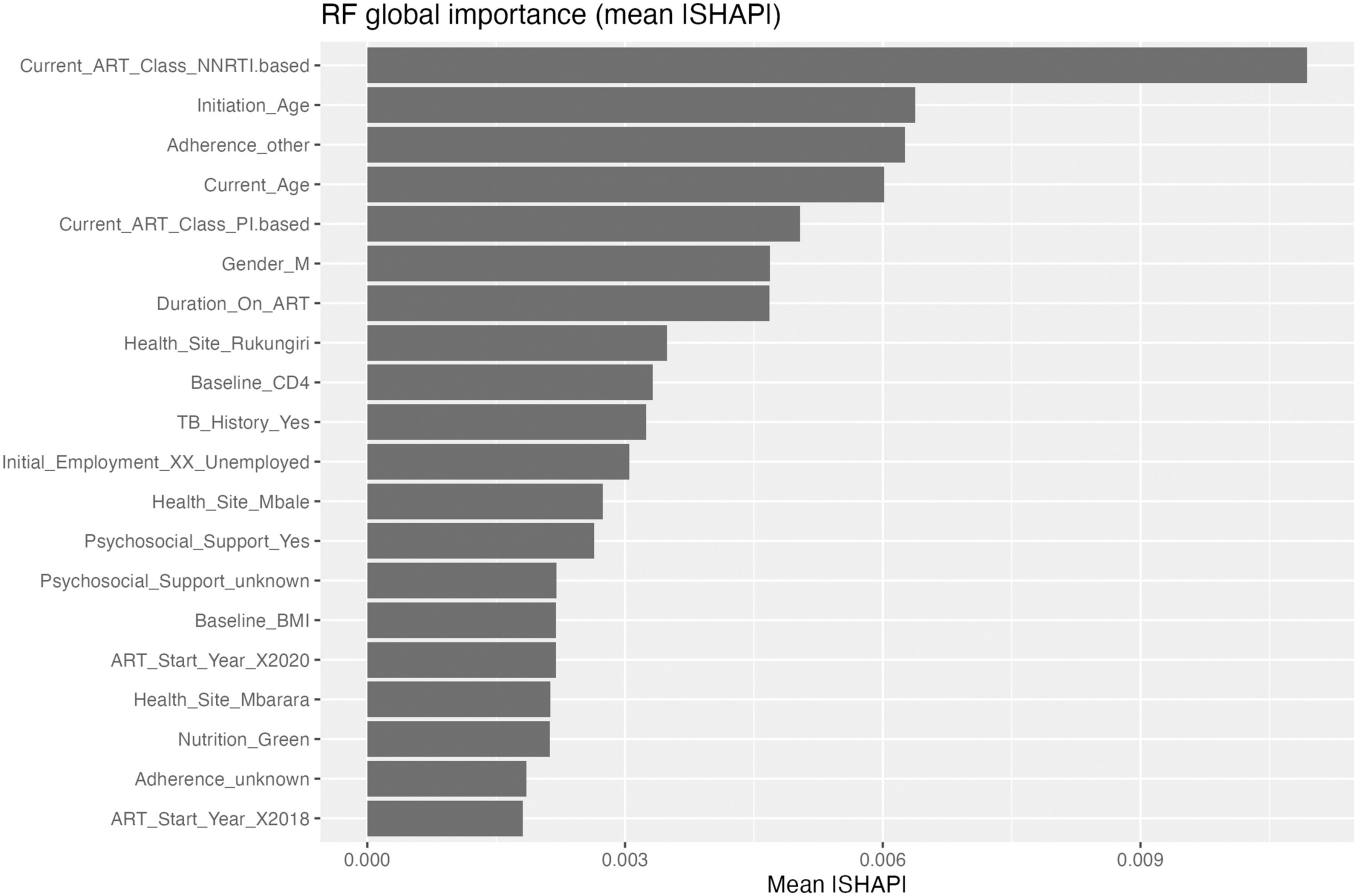
RF global feature importance by mean absolute SHAP value on the held-out test set. Mean absolute SHAP value for the top 20 predictors, derived from a random sample of 1,500 held-out test-set individuals (seed 2,601; 50 Monte Carlo replicates). Features ordered from highest to lowest mean |SHAP| contribution. Current ART class, age at ART initiation, adherence, current age, sex, and duration on ART ranked as the six highest-contributing predictors. ART, antiretroviral therapy; RF, random forest; SHAP, SHapley Additive exPlanations.

Global SHAP analysis of the RF model identified current ART class, age at ART initiation, adherence, current age, sex, and duration on ART as the six highest-ranking predictors by mean absolute SHAP value across a random sample of 1,500 held-out test-set observations (Figure 7). Additional predictors with material global importance included health site, baseline CD4 count, TB history, psychosocial support status, baseline BMI, and ART start year.

### Local patient-level explanations

Local SHAP profiles for the three highest-risk test-set individuals are shown in Supplementary Figure S1. Across all three, the two most consistently prominent contributors were age at ART initiation and undocumented baseline WHO clinical stage. The combined effect of multiple positive risk contributions (initiation age, undocumented marital status, male sex, health facility, and ART start year) was sufficient to produce net predicted probabilities substantially above the 6.5% background prevalence for all three individuals.

### Partial dependence and individual conditional expectation plots

PDP analysis identified a non-linear relationship between age at ART initiation and predicted non-suppression risk, with the steepest gradient at initiation ages below 30 and a near-flat trajectory above 45 (Supplementary Figure S2). ICE plots showed marked individual-level heterogeneity around the population average (Supplementary Figure S3).

### Covariate profile of model-identified risk strata

Individuals in the highest-risk quintile by ENET predicted risk (Q5, *n* = 1,335) differed markedly from those in the lowest-risk quintile (Q1, *n* = 1,336) across ART, demographic, and clinical domains (Table 5). The most discriminating difference was current ART class: all Q1 individuals were on an INSTI-based regimen, while Q5 comprised a mixture of INSTI- (56.3%), NNRTI- (35.1%), and PI-based (7.2%) therapy. High-risk individuals were on average 20 years younger (mean 33.0 vs. 52.9 years in Q1), initiated ART 18 years earlier in life (mean initiation age 26.3 vs. 44.3 years), and had approximately 22 fewer months on treatment (81.2 vs. 103.0 months). Male individuals were concentrated in Q5 (47.7%) relative to Q1 (21.1%). TB history was recorded in 11.2% of Q5 and fewer than 0.1% of Q1. Unmarried individuals predominated in Q5 (54.9%) relative to Q1 (36.8%). At the site level, Q1 was dominated by Mbarara (24.2%) and Rukungiri (24.1%); Q5 by Mbale (18.7%) and Tororo (13.1%).

**Table 5:** Covariate profile of the lowest-risk (Q1) and highest-risk (Q5) quintile by ENET predicted risk—held-out test set (*n* = 2,671 across both quintiles).

Characteristic	Low-risk Q1 (*n* = 1,336)	High-risk Q5 (*n* = 1,335)
Continuous—mean (SD)		
Current age, years	52.9 (11.1)	33.0 (14.1)
Age at ART initiation, years	44.3 (11.4)	26.3 (14.1)
Duration on ART, months	103.0 (25.1)	81.2 (32.7)
Categorical—% of quintile		
Sex: female	78.9%	52.3%
Sex: male	21.1%	47.7%
Current ART class: INSTI-based	100.0%	56.3%
Current ART class: NNRTI-based	0.0%	35.1%
Current ART class: PI-based	0.0%	7.2%
TB history: yes	< 0.1%	11.2%
Marital status: unmarried	36.8%	54.9%
Nutritional status: good	98.7%	84.6%
Most common sites (top 2)	Mbarara (24.2%), Rukungiri (24.1%)	Mbale (18.7%), Tororo (13.1%)

Quintiles defined by ENET-predicted probability of viral non-suppression across the held-out test set (*n* = 6,677). Q1 and Q5 each represent the extreme 20% of the predicted risk distribution (*n* ≈ 1,336 per quintile). This table is descriptive; no statistical inference is intended. Continuous variables reported as mean (standard deviation); categorical variables as percentage of quintile total. INSTI, integrase strand-transfer inhibitor; NNRTI, non-nucleoside reverse transcriptase inhibitor; PI, protease inhibitor; SD, standard deviation.

## Sensitivity analyses

### Site-held-out transportability

In leave-one-site-out validation across 11 TASO sites, PR-AUC for ENET ranged from 0.116 (Masaka, *n* = 376) to 0.304 (Jinja, *n* = 2,915), and for RF from 0.118 (Entebbe, *n* = 2,565) to 0.298 (Masindi, *n* = 2,071). At five of 11 sites (Jinja, Masindi, Mbale, Soroti, and Tororo), at least one model achieved PR-AUC above 0.25, indicating that predictive signal generalized beyond each individual site at the majority of locations. Entebbe returned the lowest PR-AUC for both models; Masaka's very low PR-AUC (0.116 for ENET) coincided with small site size and an unusually low Brier score (0.023), consistent with low local NS prevalence rather than model failure. ROC-AUC was more stable across sites, ranging from 0.439 to 0.794 for ENET and 0.639 to 0.783 for RF. Full site-level results are in Supplementary Table S3.

### Temporal robustness

When the analytic dataset was partitioned by viral load date, training on earlier records and testing on later records, PR-AUC fell for all models relative to the primary analysis: RF 0.148 (primary 0.248), ENET 0.113 (primary 0.237), XGB 0.136, LR 0.112. ROC-AUC was better preserved, ranging from 0.717 to 0.728 across models, and the relative model ranking was unchanged. The attenuation in PR-AUC is consistent with the temporal distribution shift that accompanied the NNRTI-to-INSTI policy transition across the observation window, and indicates that periodic recalibration would be needed in a prospective clinical application (Supplementary Table S5).

## Discussion

### Principal findings

This study demonstrates that routine program EMR data; without genotype data nor viral-load-derived inputs; and within the constraints of multi-site operational data quality, can support clinically meaningful non-suppression risk prediction across a large and heterogeneous national HIV program, similar to a study in Guinea (Yehadji et al., 2025). The equivalence in discrimination, calibration and clinical utility between a regularized logistic model and a random forest, confirmed by overlapping bootstrap confidence intervals rather than point-estimate comparison, is itself a substantive finding. It reframes the choice between model classes as a deployment and interpretability decision rather than a performance question, and it challenges the assumption that algorithmic complexity is necessary for useful clinical prediction (Christodoulou et al., 2019; Hu et al., 2025).

Three companion papers frame the interpretation of these findings. Our systematic review and meta-analysis with metaregression situates them within the broader East African evidence base on non-suppression predictors and prevalence (Namaganda et al., 2025). The companion descriptive cohort paper provides full program context, including the HIV treatment landscape in Uganda and corresponding policy transitions that shaped the predictor-outcome relationships observed here (Namaganda et al., 2026). The companion perspective paper makes the case for embedding risk stratification within an accountable viral load response pathway, of which this model is intended to be the clinical entry point (Namaganda, 2026, In Press).

The discrimination achieved by both models is consistent with the range reported across routine-data prediction studies in sub-Saharan Africa, and lower than that reported by single-site or research-grade studies in the same region (Bisaso et al., 2018; Mamo et al., 2023; Ngema et al., 2026). This is expected rather than concerning. Studies with higher discrimination values consistently share characteristics that are structurally absent from routine program settings: smaller, more homogeneous samples, higher predictor completeness, access to research-grade clinical measurements, or inclusion of prior viral load results as predictors. Our deliberate exclusion of viral-load-derived variables, while reducing the achievable discrimination ceiling, ensures the model can operate before a new viral load result is available; the clinically relevant scenario, (Kippen et al., 2024). A model calibrated to what routine monitoring data can deliver is more deployable, and allows generalizability, than one whose performance rests on inputs unavailable at the point of clinical use.

### ART regimen class and the INSTI transition

The dominant predictive signal in both models was current ART regimen class, with PI-based and NNRTI-based therapy identifying individuals at substantially higher non-suppression risk relative to those on INSTI-based regimens (UNAIDS, 2021). This pattern is not simply a pharmacological effect of regimen class on viral suppression. It is a composite signal that integrates treatment history, prior exposure to suboptimal regimens, accumulated resistance pressure (Namaganda et al., 2022), and the temporal sequence of the national dolutegravir rollout (Ministry of Health, 2022). Individuals still on older regimen classes a decade into the TASO program are, by definition, a selected subgroup: those who have not been transitioned, whether because of clinical contraindications, transition delays, or program-level gaps in follow-up. The elevated non-suppression odds of PI-based regimens in particular likely reflect this selection, in addition to the known resistance mechanisms associated with prolonged PI use in the context of incomplete adherence (Bircher et al., 2020; Laker et al., 2019; Mziray et al., 2020; Pau and George, 2014). The implication for program use is actionable: current ART class is a field that is already recorded, reliably, in every clinical encounter, making it the single most powerful and accessible risk signal the model uses.

### Adherence, sex, and socioeconomic vulnerability

The prominence of adherence as the second strongest predictor, and the independent contributions of male sex, unemployment, unmarried status, and undocumented psychosocial support, are consistent with the regional evidence base and with the argument that residual viraemia in the INSTI era is increasingly a function of service delivery challenges rather than regimen failures (Namaganda et al., 2026, 2025). INSTI-based regimens are highly potent and have a high barrier to resistance; in this context, non-suppression is most plausibly explained by treatment interruption or inadequate adherence support rather than pharmacological failure. The socioeconomic predictors captured in TASO's EMR; employment status, psychosocial support documentation, marital status; are fields whose independent predictive value here reinforces the case for their systematic collection at scale. Flagged individuals are not a homogeneous group: they are people whose non-suppression risk is driven by a specific, individual combination of structural and clinical factors that a multivariable model can identify but a single-variable rule cannot (Goldstein et al., 2017; Ndisha et al., 2023; Zhang et al., 2023).

### Model class equivalence and implications for deployment

The finding that a well-regularized logistic model and a random forest are statistically indistinguishable across all three evaluation domains aligns with a growing body of clinical prediction literature showing that model complexity does not reliably translate to performance gain when applied to structured tabular clinical data (Christodoulou et al., 2019). For implementers, this is practically important. Where ENET and RF perform equivalently, the ENET model offers direct interpretability through its odds ratio table: a clinician reviewing a flagged individual can understand why they were flagged, alongside lower computational requirements and a smaller implementation footprint. RF is retained as the primary model because of its pre-specified SHAP explainability objectives; the two frameworks provide complementary evidence that converges on the same predictors. The exclusion of XGB, despite its highest ROC-AUC, is a concrete illustration of why calibration must be assessed alongside discrimination: a model whose predicted probabilities cannot be trusted cannot be used to set meaningful clinical thresholds, regardless of its ranking on a discrimination metric (Van Calster et al., 2019).

### Calibration, clinical utility, and the case for threshold-based deployment

The practical significance of this study lies not in the discrimination metrics alone but in the convergence of calibration and decision curve analysis in supporting threshold-based prioritization. A calibrated model means that predicted probabilities are actionable: a program can choose a threshold, apply it, and have confidence that the yield of non-suppressed individuals among those flagged reflects the stated PPV (Vickers and Elkin, 2006). The capacity-first operating point directly addresses the question a clinic manager asks: if I can only intensify review for a small proportion of my caseload each month, who should those individuals be? Both models demonstrate that a 5% flag rate would concentrate over 30% of non-suppressed individuals into under 5% of the follow-up burden: a prioritization gain that cannot be achieved by any single clinical variable and that is transparently quantified before deployment.

### Site transportability and what it means for implementation

The variation in predictive performance across TASO sites is an insightful finding, not a limitation to be minimized. It reflects real heterogeneity in site size, local NS prevalence, and data capture completeness that would affect any deployed tool. At sites where leave-one-site-out PR-AUC was low, the explanation was more often low local NS prevalence than model failure: a distinction that matters for implementation, because the appropriate response is site-specific threshold calibration rather than model abandonment. The substantially more stable ROC-AUC across sites reinforces the interpretive limitation of ROC-AUC in imbalanced outcomes: it would suggest uniform performance across all sites, obscuring the prevalence-related variation that is precisely what a deployment framework needs to account for. Any implementation plan should treat site-level performance monitoring as a routine operational requirement, not an afterthought (Debray et al., 2013).

### Translating prediction into practice

The intended function of this tool is a decision-support input, not a replacement for clinical judgement (Shortliffe and Sepúlveda, 2018). Its value is in reliably identifying a manageable subset of individuals whose risk profile warrants targeted engagement before their next viral load result arrives, within real staffing and capacity constraints. The predictor profile of the high-risk quintile: younger individuals, on older regimens, with documented adherence gaps and socioeconomic vulnerability, describes people who are reachable through targeted differentiated service delivery, intensified counseling, and psychosocial support: interventions that already exist within the TASO framework. The model's role is to direct those interventions efficiently. All associations in this study are predictive, derived from observational program data, and should be interpreted accordingly.

## Strengths and Limitations

### Strengths

This study has several features that support its program relevance and methodological integrity. The cohort is large, multi-site, and spans a decade of service delivery across Uganda's national program, providing a scale and heterogeneity that directly represents the operational context for deployment. Predictors were restricted to routinely captured, deployment-feasible variables, and viral-load-derived predictors were explicitly excluded to prevent leakage and to produce a realistic estimate of routine-data operational performance. Model evaluation spanned three complementary domains: discrimination, calibration, and clinical utility; with bootstrap confidence intervals throughout, supporting comparative statements rather than point-estimate comparisons. Two pre-specified operating points map continuous predicted probabilities directly to program workflow decisions. A two-layer explainability framework: clinician-interpretable odds ratios for ENET and SHAP global and local importance for RF; supports transparency and practical uptake. Site-held-out and temporal analyses characterize the model's transportability and its behavior under the treatment policy transition that occurred across the observation window.

### Limitations

Five limitations should be stated plainly. First, the outcome is non-suppression at the most recent viral load rather than confirmed virological failure; repeat confirmatory testing and consistently documented adherence counseling completion records were not uniformly available, so the target is the most consistently observable program risk state, not the formal failure definition. Second, the 38.6% of individuals in the parent cohort without a recorded viral load were excluded from modeling. If exclusion correlates with non-suppression risk; for example, among individuals with disrupted care or lost to follow-up; the training population may underrepresent the highest-risk individuals and model performance in that subgroup is unknown. Third, internal validation: including site-held-out and temporal sensitivity analyses, does not substitute for prospective evaluation in a live workflow or for independent external validation in cohorts outside the TASO network; performance in real program conditions remains to be established. Fourth, sparsely recorded predictors (baseline CD4 count, available in 35.1% of training records; baseline BMI in 24.7%) introduce residual uncertainty; if the probability of a value being recorded is related to patient risk status, missingness indicators can partially but not fully compensate. Fifth, the model decays under temporal distribution shift: PR-AUC fell substantially in the temporal split analysis (RF 0.148 vs. primary 0.248), consistent with the NNRTI-to-INSTI transition creating a different predictor-outcome relationship across the observation window. Any deployment strategy would require periodic performance monitoring and structured recalibration; triggered no less than annually, or upon material changes in local ART class distribution; as treatment policies continue to evolve. Sixth, the odds ratio of 11.07 for PI-based regimens should be interpreted as a composite clinical signal that integrates current pharmacological regimen with prior treatment history, selection into a salvage therapy subgroup following NNRTI failure, accumulated resistance pressure, and transition delays—rather than as a direct pharmacological effect of PI therapy. As a predictor in a deployment tool, PI-based regimen status is informative precisely because it captures this history; clinicians reviewing a flagged individual should interpret this variable in its full clinical context. Seventh, the psychosocial and socioeconomic predictors that contribute meaningfully to model performance: adherence category, psychosocial support status, employment status, and nutritional status, are relatively captured in TASO's structured care model but are not routinely recorded in most Ministry of Health-run public HIV facilities, where the majority of people living with HIV in Uganda receive care. Full model replication in public-sector settings would require either comparable data infrastructure or re-training on locally available variables. Generalizability to MoH facilities cannot be assumed and would need to be prospectively assessed in a setting with appropriate data collection. Eighth, while clinic-level heterogeneity was addressed empirically through inclusion of health site as a predictor variable in all models, reporting of between-site odds ratios in the ENET logistic regression, and leave-one-site-out cross-validation, an intracluster correlation coefficient (ICC) from a null multilevel model was not estimated. For the prediction models, site inclusion as a predictor and the leave-one-site-out validation address the operationally relevant questions of site adjustment and cross-site transportability. For the ENET refit logistic regression, within-clinic correlation could affect the precision of standard error estimates. Formal multilevel modeling of predictor-outcome associations in this dataset represents a direction for future work.

## Conclusion

We developed and internally validated explainable, routine EMR-based risk stratification models for viral non-suppression prediction in the TASO Uganda cohort. Elastic net regularized logistic regression and random forest achieved comparable, clinically meaningful performance across discrimination, calibration, and decision curve analysis—with bootstrap confidence intervals confirming that neither model statistically outperformed the other, or standard logistic regression, on PR-AUC alone. Model selection was appropriately grounded in calibration and clinical utility rather than discrimination rank. The dominant drivers of non-suppression—current ART regimen class, poor adherence, TB co-infection, male sex, and markers of socioeconomic vulnerability—are consistent with our companion systematic review and the broader East African evidence base, and remain directly actionable within program frameworks. At the capacity-first operating threshold, both models achieved 4.7- to 5.2-fold enrichment of non-suppressed individuals in the top 5% of predicted risk, with NPV exceeding 94.8% among those not flagged. These findings support the use of routine program EMR data for risk-stratified HIV care in Uganda and similar resource-limited settings; prospective external validation and careful workflow integration are the essential next steps before clinical deployment.

## Data Availability

The data analyzed in this study is subject to the following licenses/restrictions: The TASO programme data are de-identified but not publicly available — they are held by TASO Uganda under institutional data governance and require a formal data sharing agreement and ethical approval to access. The patient-level data are not included. Only aggregated results, summary statistics, and model outputs appear in the manuscript and Supplementary material. Requests to access these datasets should be directed to Simple Ouma, oumas@tasouganda.org.
