# Supplementary material for: Predicting HIV viral non-suppression in Uganda: development and validation of machine learning and risk stratification models using routine EMR data
Source: Front Artif Intell. 2026 Jul 16;9:1869992. doi: 10.3389/frai.2026.1869992 (PMC13422409; doi:10.3389/frai.2026.1869992)
Supplement: Supplementary file 1 [file Data_Sheet_1.docx]

**Supplementary materials**

Predicting viral non-suppression using routine EMR data in Uganda: development and validation of machine learning and statistical risk stratification models in the TASO HIV cohort (2014–2024)

## Contents

Supplementary File S1. TRIPOD-AI reporting checklist (attached separately)

Supplementary Table S1. Predictor availability in the training set

Supplementary Table S2. Predictor distributions in the training set

Supplementary Table S3. Site-level leave-one-site-out cross-validation results

Supplementary Table S4. Full ENET refit odds ratio table

Supplementary Table S5. Model performance under temporal split

Supplementary Figure S1. Local RF SHAP profiles (three highest-risk individuals)

Supplementary Figure S2. Partial dependence plot — age at ART initiation

Supplementary Figure S3. ICE plots — age at ART initiation

## Supplementary File S1. TRIPOD-AI reporting checklist.

The completed TRIPOD-AI reporting checklist for this prediction model development and validation study is attached separately. TRIPOD-AI reporting standards are described in: Collins GS et al. BMJ 2024;385:e078378.

## Supplementary Table S1. Availability of Feature Set A predictors in the training set (n = 26,707).

| **Predictor** | **Missing, n** | **% Missing** | **Availability tier** |
| --- | --- | --- | --- |
| **SPARSE (>20% MISSING)** |  |  |  |
| Baseline BMI (kg/m²) | 20,113 | 75.3% | Sparse (>20% missing) |
| Baseline CD4 count (cells/µL) | 17,331 | 64.9% | Sparse (>20% missing) |
| **MODERATE (5–20% MISSING)** |  |  |  |
| TB history | 4,264 | 16.0% | Moderate (5–20% missing) |
| Education level | 3,618 | 13.5% | Moderate (5–20% missing) |
| Adherence category | 2,882 | 10.8% | Moderate (5–20% missing) |
| Employment status | 2,820 | 10.6% | Moderate (5–20% missing) |
| Baseline WHO stage | 2,433 | 9.1% | Moderate (5–20% missing) |
| Diabetes | 1,899 | 7.1% | Moderate (5–20% missing) |
| Psychosocial support status | 1,822 | 6.8% | Moderate (5–20% missing) |
| Hypertension | 1,654 | 6.2% | Moderate (5–20% missing) |
| **ROUTINE (≤5% MISSING)** |  |  |  |
| Marital status | 989 | 3.7% | Routine (≤5% missing) |
| Baseline ART class | 656 | 2.5% | Routine (≤5% missing) |
| Nutritional status | 509 | 1.9% | Routine (≤5% missing) |
| Age at ART initiation (years) | 7 | 0.0% | Routine (≤5% missing) |
| Current ART class | 2 | 0.0% | Routine (≤5% missing) |
| Health site | 0 | 0% | Routine (≤5% missing) |
| Sex | 0 | 0% | Routine (≤5% missing) |
| Current age (years) | 0 | 0% | Routine (≤5% missing) |
| ART start year | 0 | 0% | Routine (≤5% missing) |
| Duration on ART (months) | 0 | 0% | Routine (≤5% missing) |
| *Training set, n = 26,707. Missing n estimated as % × 26,707, rounded to nearest integer. Predictors classified as routine (≤5% missing), moderate (5–20%), or sparse (>20%). Binary missingness indicators were created for all numeric predictors prior to imputation; nominal predictors with missing values were assigned a dedicated ‘unknown’ level. ART = antiretroviral therapy; BMI = body mass index; WHO = World Health Organization.* | | | |

## Supplementary Table S2. Predictor distributions in the training set (n = 26,707).

| **Predictor** | **Category / Level** | **n (observed)** | **Distribution** | **% Missing** |
| --- | --- | --- | --- | --- |
| **DEMOGRAPHIC / ADMINISTRATIVE** |  |  |  |  |
| **Sex** |  |  |  | **0%** |
|  | F | 16,452 | 61.6% |  |
|  | M | 10,255 | 38.4% |  |
| Current age (years) | Median [IQR]; range | 26,707 | 41.5 [33.5–50.5]; 1.3–99.2 | 0% |
| Age at ART initiation (years) | Median [IQR]; range | 26,700 | 34.6 [27–43.2]; 0–91.4 | 0% |
| ART start year | Median [IQR]; range | 26,707 | 2017 [2015–2019]; 2014–2024 | 0% |
| **Health site** |  |  |  | **0%** |
|  | Entebbe | 2,565 | 9.6% |  |
|  | Gulu | 684 | 2.6% |  |
|  | Jinja | 2,915 | 10.9% |  |
|  | Masaka | 376 | 1.4% |  |
|  | Masindi | 2,071 | 7.8% |  |
|  | Mbale | 3,320 | 12.4% |  |
|  | Mbarara | 2,931 | 11% |  |
|  | Mulago | 4,152 | 15.5% |  |
|  | Rukungiri | 2,852 | 10.7% |  |
|  | Soroti | 1,229 | 4.6% |  |
|  | Tororo | 3,612 | 13.5% |  |
| **TREATMENT HISTORY** |  |  |  |  |
| Duration on ART (months) | Median [IQR]; range | 26,707 | 84.8 [61.1–113.8]; 0.4–131.3 | 0% |
| **Baseline ART class** |  |  |  | **2.5%** |
|  | INSTI-based | 6,959 | 26.7% |  |
|  | NNRTI-based | 17,591 | 67.5% |  |
|  | PI-based | 301 | 1.2% |  |
|  | Unknown | 1,200 | 4.6% |  |
| **Current ART class** |  |  |  | **0%** |
|  | INSTI-based | 24,179 | 90.5% |  |
|  | NNRTI-based | 2,064 | 7.7% |  |
|  | PI-based | 383 | 1.4% |  |
|  | Unknown | 79 | 0.3% |  |
| **IMMUNO-CLINICAL** |  |  |  |  |
| Baseline CD4 count (cells/µL) | Median [IQR]; range | 9,376 | 370 [209–531]; 0–44943 | 64.9% |
| **Baseline WHO stage** |  |  |  | **9.1%** |
|  | WHO_1 | 6,392 | 26.3% |  |
|  | WHO_2 | 15,389 | 63.4% |  |
|  | WHO_3 | 861 | 3.5% |  |
|  | WHO_4 | 235 | 1% |  |
|  | Unknown | 1,397 | 5.8% |  |
| **CLINICAL / NUTRITIONAL** |  |  |  |  |
| Baseline BMI (kg/m²) | Median [IQR]; range | 6,594 | 21.9 [19–25.8]; 0.4–4297.5 | 75.3% |
| **Nutritional status** |  |  |  | **1.9%** |
|  | Unknown | 480 | 1.8% |  |
|  | Green | 24,798 | 94.7% |  |
|  | Red | 163 | 0.6% |  |
|  | Yellow | 757 | 2.9% |  |
| **COMORBIDITIES** |  |  |  |  |
| **TB history** |  |  |  | **16%** |
|  | No | 21,460 | 95.6% |  |
|  | Yes | 983 | 4.4% |  |
| **Diabetes** |  |  |  | **7.1%** |
|  | No | 24,324 | 98% |  |
|  | Yes | 484 | 2% |  |
| **Hypertension** |  |  |  | **6.2%** |
|  | No | 23,652 | 94.4% |  |
|  | Yes | 1,401 | 5.6% |  |
| **PSYCHOSOCIAL / SOCIOECONOMIC** |  |  |  |  |
| **Adherence category** |  |  |  | **10.8%** |
|  | Fair | 196 | 0.8% |  |
|  | Good | 23,383 | 98.1% |  |
|  | Poor | 227 | 1% |  |
|  | Unknown | 19 | 0.1% |  |
| **Marital status** |  |  |  | **3.7%** |
|  | Married | 14,035 | 54.6% |  |
|  | Unknown | 401 | 1.6% |  |
|  | Unmarried | 11,282 | 43.9% |  |
| **Education level** |  |  |  | **13.5%** |
|  | None | 2,745 | 11.9% |  |
|  | Primary_or_Lower | 12,680 | 54.9% |  |
|  | Secondary | 5,944 | 25.7% |  |
|  | Higher_Institute | 1,491 | 6.5% |  |
|  | Unknown | 229 | 1% |  |
| **Employment status** |  |  |  | **10.6%** |
|  | Regular_Income | 4,509 | 18.9% |  |
|  | Irregular_Income | 13,028 | 54.5% |  |
|  | Unemployed | 5,244 | 22% |  |
|  | Unknown | 1,106 | 4.6% |  |
| **Psychosocial support status** |  |  |  | **6.8%** |
|  | No | 1,499 | 6% |  |
|  | Yes | 23,386 | 94% |  |
| *Training set, n = 26,707. For categorical predictors, % is calculated among non-missing observations. For continuous predictors, median [IQR] and range are reported. Missingness rates cross-reference Supplementary Table S1. ART = antiretroviral therapy; BMI = body mass index; IQR = interquartile range; WHO = World Health Organization.* | | | | |

## Supplementary Table S3. Leave-one-site-out cross-validation performance by TASO site — ENET and RF models.

| **Site** | **n** | **ENET PR-AUC** | **ENET ROC-AUC** | **ENET Brier** | **RF PR-AUC** | **RF ROC-AUC** | **RF Brier** |
| --- | --- | --- | --- | --- | --- | --- | --- |
| Entebbe | 2,565 | 0.135 | 0.676 | 0.065 | 0.118 | 0.639 | 0.063 |
| Gulu | 684 | 0.185 | 0.668 | 0.093 | 0.249 | 0.709 | 0.088 |
| Jinja | 2,915 | 0.304 | 0.781 | 0.052 | 0.264 | 0.783 | 0.054 |
| Masaka | 376 | 0.116 | 0.439 | 0.023 | 0.209 | 0.655 | 0.024 |
| Masindi | 2,071 | 0.288 | 0.746 | 0.064 | 0.298 | 0.751 | 0.064 |
| Mbale | 3,320 | 0.301 | 0.736 | 0.074 | 0.290 | 0.704 | 0.075 |
| Mbarara | 2,931 | 0.220 | 0.765 | 0.038 | 0.197 | 0.760 | 0.038 |
| Mulago | 4,152 | 0.248 | 0.751 | 0.050 | 0.243 | 0.739 | 0.051 |
| Rukungiri | 2,852 | 0.211 | 0.794 | 0.085 | 0.118 | 0.723 | 0.078 |
| Soroti | 1,229 | 0.288 | 0.769 | 0.069 | 0.297 | 0.759 | 0.069 |
| Tororo | 3,612 | 0.254 | 0.710 | 0.059 | 0.264 | 0.687 | 0.060 |
| *Leave-one-site-out cross-validation: for each site in turn, ENET and RF were trained on data from all remaining 10 sites using the primary best-performing hyperparameter configuration, then evaluated on the held-out site. This analysis assesses transportability within the TASO network; it does not constitute full external validation. PR-AUC null equals local non-suppression prevalence (varies by site). Brier null = p(1−p) where p = site-specific NS prevalence. ENET = elastic net regularised logistic regression; RF = random forest; PR-AUC = precision-recall area under the curve; ROC-AUC = receiver operating characteristic area under the curve.* | | | | | | | |

## Supplementary Table S4. Full elastic net refit odds ratio table — all 43 non-zero predictor terms (held-out test set).

| **Predictor / Level** | **OR** | **95% CI** | **p-value** |
| --- | --- | --- | --- |
| **TREATMENT HISTORY** |  |  |  |
| Current ART class: PI-based | 11.07 | 8.68–14.08 | <0.001 |
| Current ART class: NNRTI-based | 4.80 | 4.10–5.61 | <0.001 |
| Current ART class: other/unknown | 3.64 | 1.85–6.64 | <0.001 |
| Baseline ART class: NNRTI-based | 1.01 | 0.85–1.21 | 0.898 |
| Baseline ART class: unknown (missing-imputed) | 0.76 | 0.48–1.16 | 0.224 |
| Baseline ART class: Unknown (recorded level) | 1.16 | 0.88–1.52 | 0.279 |
| Duration on ART (per month) | 0.98 | ≤0.99 | 0.751 |
| ART start year: 2015 | 0.92 | 0.75–1.12 | 0.386 |
| ART start year: 2017 | 0.89 | 0.74–1.07 | 0.216 |
| ART start year: 2018 | 1.14 | 0.98–1.33 | 0.083 |
| ART start year: 2020 | 1.17 | 0.97–1.41 | 0.101 |
| ART start year: pre-2015/unknown | 0.50 | 0.23–0.95 | 0.051 |
| **DEMOGRAPHIC / ADMINISTRATIVE** |  |  |  |
| Sex: male | 1.62 | 1.45–1.80 | <0.001 |
| Current age (per year) | 1.17 | 1.00–≥ | 0.833 |
| Age at ART initiation (per year) | 0.83 | ≤0.97 | 0.806 |
| Health site: Mbale | 1.65 | 1.39–1.94 | <0.001 |
| Health site: Gulu | 0.74 | 0.16–2.44 | 0.657 |
| Health site: Masaka | 0.61 | 0.28–1.18 | 0.177 |
| Health site: Masindi | 1.08 | 0.88–1.33 | 0.433 |
| Health site: Mbarara | 0.58 | 0.46–0.71 | <0.001 |
| Health site: Rukungiri | 0.69 | 0.53–0.87 | 0.003 |
| Health site: Tororo | 0.70 | 0.16–2.14 | 0.582 |
| **IMMUNO-CLINICAL** |  |  |  |
| Baseline CD4 count (per cell/µL) | 1.00 | 1.00–1.00 | 0.203 |
| Baseline WHO stage: 2 | 0.95 | 0.84–1.07 | 0.417 |
| Baseline WHO stage: 3 | 1.11 | 0.83–1.46 | 0.470 |
| **CLINICAL / NUTRITIONAL** |  |  |  |
| Nutritional status: good (green) | 0.83 | 0.58–1.23 | 0.340 |
| Nutritional status: at-risk (yellow) | 1.13 | 0.73–1.78 | 0.590 |
| Nutritional status: other | 1.33 | 0.73–2.39 | 0.351 |
| Nutritional status: unknown | 1.33 | 0.83–2.15 | 0.243 |
| **COMORBIDITIES** |  |  |  |
| TB history: yes | 2.08 | 1.67–2.57 | <0.001 |
| TB history: unknown | 1.88 | 0.61–8.25 | 0.325 |
| Diabetes: yes | 0.59 | 0.41–0.84 | 0.004 |
| Hypertension: yes | 0.85 | 0.63–1.13 | 0.276 |
| Hypertension: unknown | 1.14 | 0.86–1.51 | 0.353 |
| **PSYCHOSOCIAL / SOCIOECONOMIC** |  |  |  |
| Adherence: poor/other | 7.71 | 6.18–9.59 | <0.001 |
| Marital status: unmarried | 1.16 | 1.04–1.30 | 0.009 |
| Marital status: unknown | 1.29 | 0.96–1.70 | 0.082 |
| Employment status: unemployed | 1.25 | 1.09–1.43 | 0.001 |
| Employment status: unknown | 0.80 | 0.59–1.05 | 0.120 |
| Education: primary or lower | 1.11 | 0.99–1.24 | 0.062 |
| Education: other/unknown | 0.80 | 0.40–1.44 | 0.482 |
| Psychosocial support: yes | 0.94 | 0.75–1.18 | 0.562 |
| Psychosocial support: undocumented | 1.45 | 1.05–1.99 | 0.022 |
| *Odds ratios (OR) and 95% confidence intervals (CI) are from an unpenalised logistic regression refit using only the 43 predictors with non-zero ENET coefficients at the selected penalty value; regularised ENET coefficients are shrunk toward zero and cannot be directly exponentiated. Reference categories: current ART class = INSTI-based; sex = female; TB history = no; adherence = good; nutritional status = poor/red; baseline WHO stage = 1; marital status = married/partnered; employment = employed; education = secondary or higher; hypertension = no; diabetes = no; psychosocial support = no; baseline ART class = INSTI-based; health site = Entebbe; ART start year = 2016. ≤/≥ = confidence interval boundary could not be estimated (near-separation). ART = antiretroviral therapy; CI = confidence interval; ENET = elastic net regularised logistic regression; INSTI = integrase strand-transfer inhibitor; NNRTI = non-nucleoside reverse transcriptase inhibitor; OR = odds ratio; PI = protease inhibitor; WHO = World Health Organization.* | | | |

## Supplementary Table S5. Model performance under temporal split sensitivity analysis.

| **Model** | **Temporal PR-AUC** | **Temporal ROC-AUC** | **Primary PR-AUC (95% CI)** | **Primary ROC-AUC (95% CI)** |
| --- | --- | --- | --- | --- |
| Random forest (RF) | 0.148 | 0.717 | 0.248 (0.207–0.291) | 0.758 (0.732–0.780) |
| Extreme gradient boosting (XGB) | 0.136 | 0.728 | — | — |
| Elastic net (ENET) | 0.113 | 0.722 | 0.237 (0.198–0.279) | 0.750 (0.726–0.772) |
| Logistic regression (LR) | 0.112 | 0.719 | — | — |
| *Temporal split: the analytic dataset was partitioned by viral load date (80th percentile as threshold), with earlier records assigned to training and later records to testing. The same preprocessing pipeline and best-performing hyperparameters from the primary analysis were applied. Viral load date was used only for partitioning and was not included as a predictor. Primary analysis PR-AUC and ROC-AUC with bootstrap 95% CIs (2,000 replicates, seed 2601) are shown for RF and ENET only; bootstrap CIs were not computed for LR and XGB in the primary analysis. PR-AUC null = non-suppression prevalence in the temporal test set. ENET = elastic net regularised logistic regression; LR = logistic regression; PR-AUC = precision-recall area under the curve; RF = random forest; ROC-AUC = receiver operating characteristic area under the curve; XGB = extreme gradient boosting.* | | | | |

## Supplementary Figure S1. Local RF SHAP profiles for the three highest-risk individuals in the held-out test set.


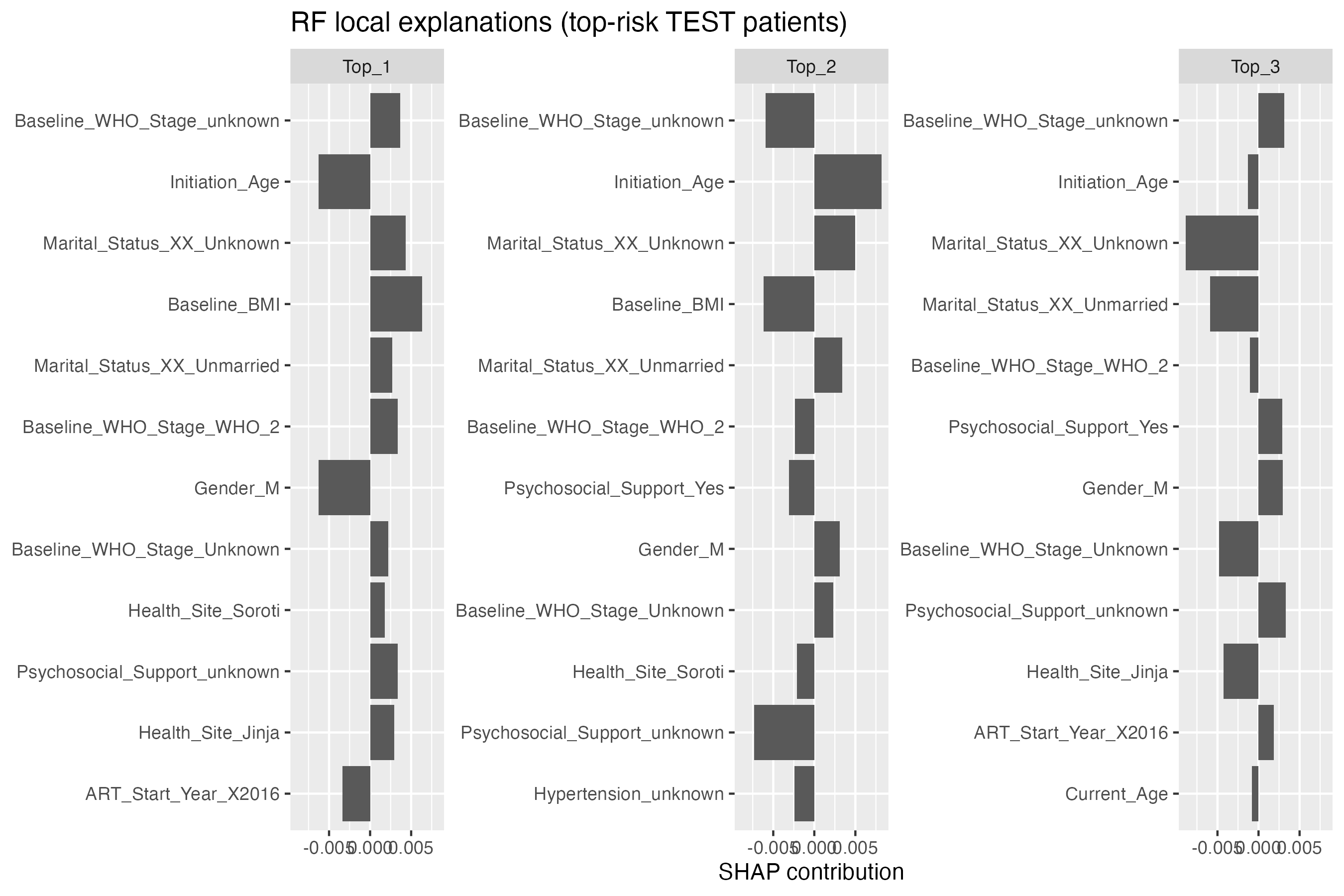


Supplementary Figure S1. SHAP waterfall plots for the three test-set individuals with the highest RF-predicted non-suppression probability. Each bar shows the SHAP contribution of one predictor to the deviation from the baseline log-odds. Red bars increase predicted risk; blue bars decrease it. The two most consistently prominent contributors across all three individuals were age at ART initiation and undocumented baseline WHO clinical stage. ART = antiretroviral therapy; RF = random forest; SHAP = SHapley Additive exPlanations; WHO = World Health Organization.

## Supplementary Figure S2. Partial dependence of RF-predicted non-suppression risk on age at ART initiation.


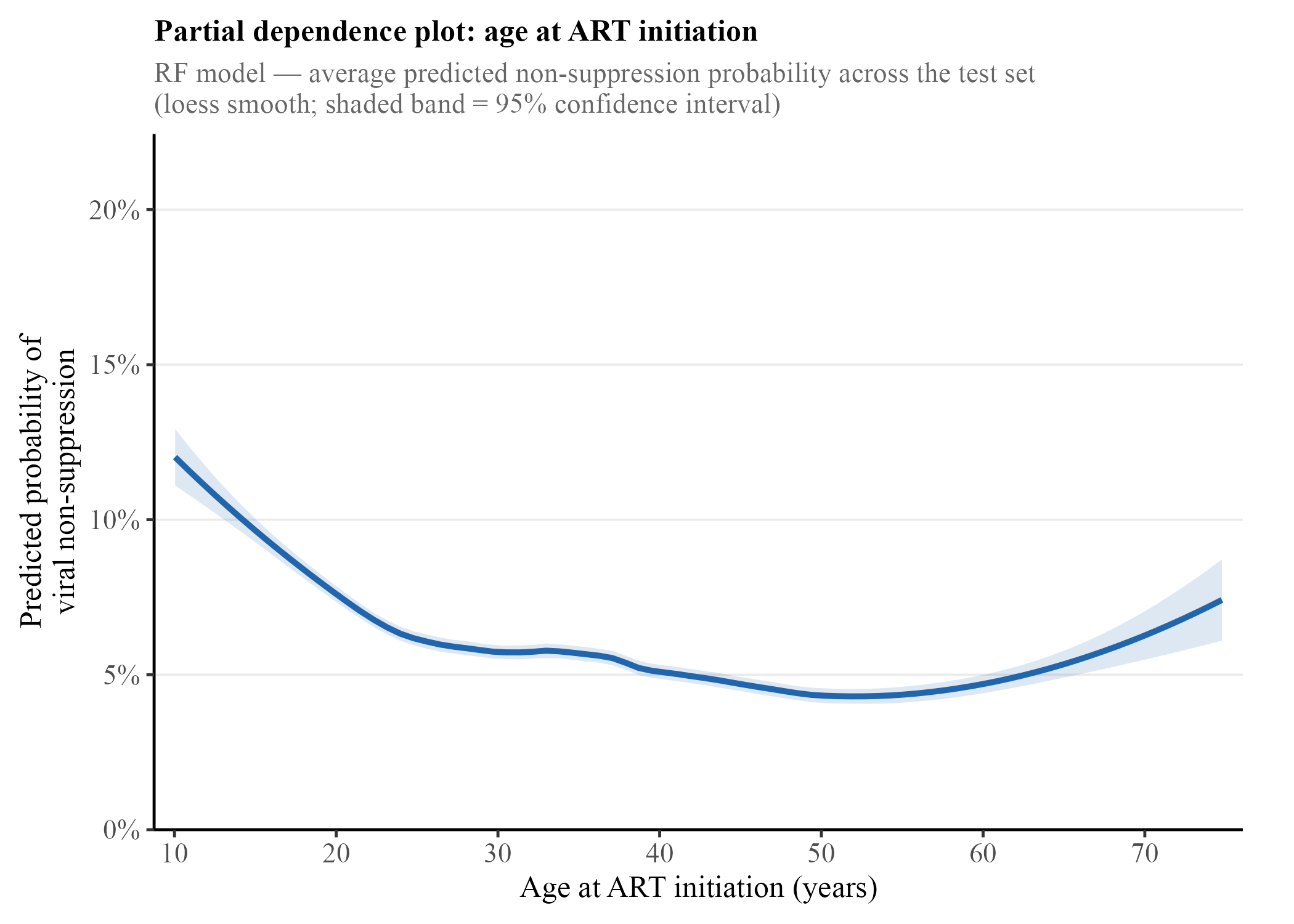


Supplementary Figure S2. RF-predicted non-suppression probability as a function of age at ART initiation, estimated on the held-out test set (n = 6,677). Points show individual RF-predicted probabilities; the solid line and shaded band show a loess smooth (span = 0.4) with 95% pointwise confidence interval. The relationship is non-linear: predicted risk is highest at initiation ages below 30, with the steepest gradient below age 20, and approaches a near-flat trajectory above age 45. RF = random forest.

## Supplementary Figure S3. Individual conditional expectation (ICE) plots for age at ART initiation.


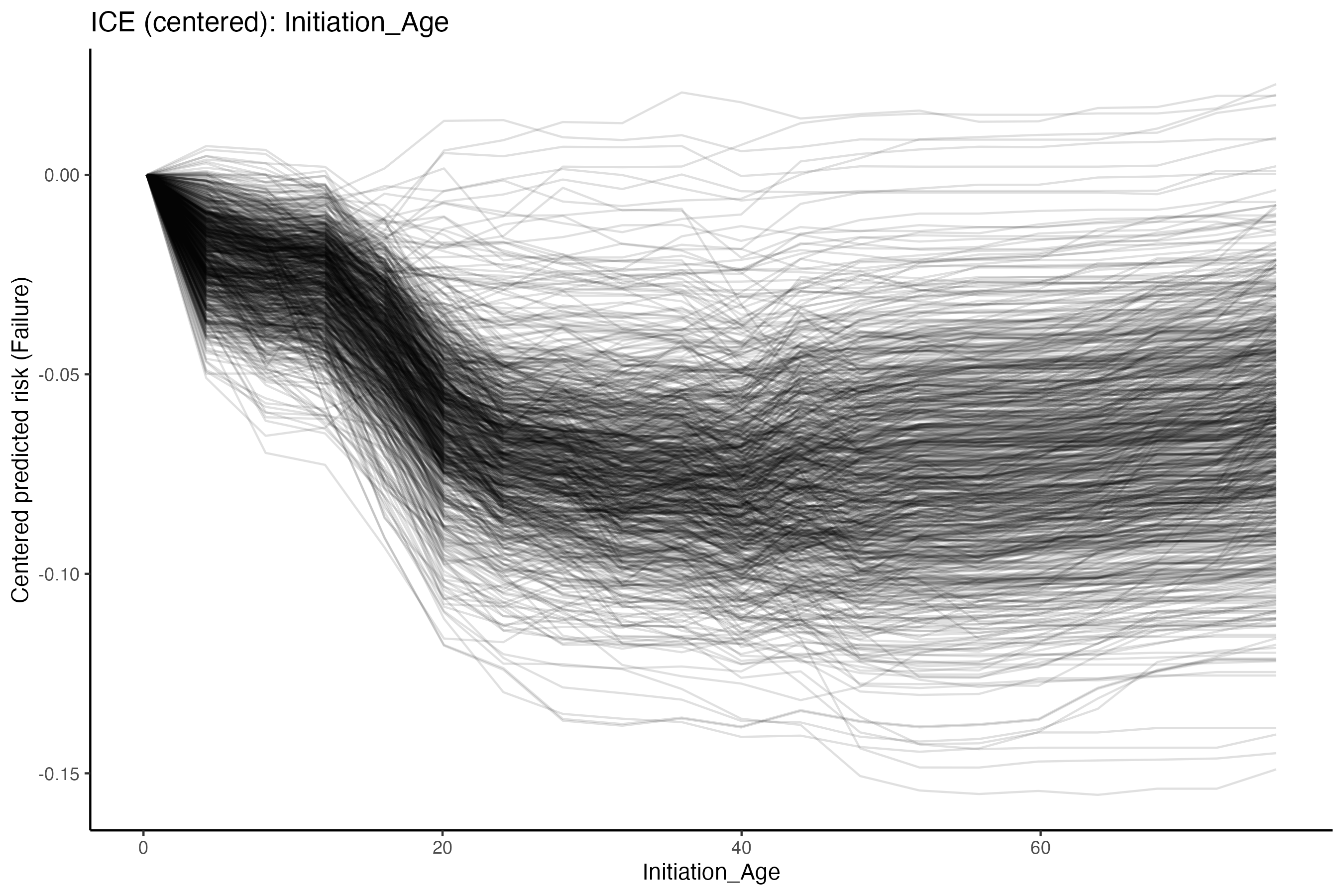


Supplementary Figure S3. Individual conditional expectation (ICE) plots showing the predicted non-suppression probability for each test-set individual across the observed range of age at ART initiation, holding all other predictors at their observed values. Each line represents one individual. The bold line is the population average (partial dependence). Marked heterogeneity across individual trajectories indicates that the relationship between initiation age and predicted risk varies substantially depending on the individual’s other predictor values. RF = random forest.
